# Whole-genome analysis of Malawian *Plasmodium falciparum* isolates identifies possible targets of allele-specific immunity to clinical malaria

**DOI:** 10.1371/journal.pgen.1009576

**Published:** 2021-05-25

**Authors:** Zalak Shah, Myo T. Naung, Kara A. Moser, Matthew Adams, Andrea G. Buchwald, Ankit Dwivedi, Amed Ouattara, Karl B. Seydel, Don P. Mathanga, Alyssa E. Barry, David Serre, Miriam K. Laufer, Joana C. Silva, Shannon Takala-Harrison

**Affiliations:** 1 Center for Vaccine Development and Global Health, University of Maryland School of Medicine, Baltimore, Maryland, United States of America; 2 Population Health and Immunity Division, Walter Eliza Hall of Medical Institute for Medical Research, Parkville, Victoria, Australia; 3 Department of Medical Biology, University of Melbourne, Carlton, Victoria, Australia; 4 School of Medicine, Deakin University, Geelong, Victoria, Australia; 5 Institute for Genome Sciences, University of Maryland School of Medicine, Baltimore, Maryland, United States of America; 6 Department of Osteopathic Medical Specialties, College of Osteopathic Medicine, Michigan State University, East Lansing, Michigan, United States of America; 7 Blantyre Malaria Project, University of Malawi College of Medicine, Blantyre, Malawi; 8 University of Malawi College of Medicine, Malaria Alert Centre, Blantyre, Malawi; 9 Disease Elimination and Maternal and Child Health, Burnet Institute, Melbourne, Victoria, Australia; London School of Hygiene and Tropical Medicine, UNITED KINGDOM

## Abstract

Individuals acquire immunity to clinical malaria after repeated *Plasmodium falciparum* infections. Immunity to disease is thought to reflect the acquisition of a repertoire of responses to multiple alleles in diverse parasite antigens. In previous studies, we identified polymorphic sites within individual antigens that are associated with parasite immune evasion by examining antigen allele dynamics in individuals followed longitudinally. Here we expand this approach by analyzing genome-wide polymorphisms using whole genome sequence data from 140 parasite isolates representing malaria cases from a longitudinal study in Malawi and identify 25 genes that encode possible targets of naturally acquired immunity that should be validated immunologically and further characterized for their potential as vaccine candidates.

## Author summary

Each year, there are hundreds of millions of cases of malaria resulting in several hundred thousand deaths. In malaria-endemic areas with high transmission, individuals experience malaria illness multiple times during their lifetimes, and after many infections, develop immunity that prevents symptoms. The proteins targeted by acquired immunity are not fully known. Understanding which proteins are targets of protective immune responses may aid in development of a malaria vaccine. Here, we used whole-genome sequence data from malaria parasites collected from symptomatic individuals in Malawi to identify targets of malaria immunity based on the frequency of different sequence variants observed in people with different levels of immunity and in individuals over time. Using the combined results of these approaches, we identified genes encoding 25 proteins that may be targets of clinical immunity to malaria that will be further investigated in future studies for their potential as vaccine candidate antigens.

## Introduction

Despite recent progress in reducing the burden of malaria, this disease remains a leading cause of mortality worldwide, resulting in an estimated 409,000 deaths in 2019 [1]. In areas with high transmission of *Plasmodium falciparum*, individuals develop immunity to malaria [2]. This immunity does not provide sterile protection against all infections, but decreases the risk of clinical disease, and increases with age as individuals are repeatedly exposed to the parasite [3,4]. This age-related pattern of immunity to disease is thought to reflect the need for a repertoire of immune responses to multiple alleles in diverse parasite antigens [2,3,5,6]. The extensive genetic diversity in *P*. *falciparum* surface antigens is thought to have evolved over millennia as a means of parasite immune evasion [7]. Allele-specific immune responses have been demonstrated for several parasite antigens [8–15]. In previous work, we examined parasite alleles in repeated infections occurring in individuals followed longitudinally and identified specific polymorphic sites within parasite surface antigens (i.e. AMA1 and MSP1) where amino acid changes were associated with immune escape and increased risk of disease, consistent with allele-specific acquisition of immunity to these antigens [16,17]. Furthermore, malaria subunit vaccines based on a single antigen allele have displayed greater efficacy against parasites with alleles matching the vaccine strain compared to the diverse alleles observed in natural parasite populations [17–20]. Such allele-specific vaccine efficacy could lead to poor overall vaccine efficacy when the vaccine target allele is at low frequency in the parasite population and could result in selection of non-vaccine alleles capable of vaccine escape [18]. Overcoming this scenario may require the design of a multivalent malaria vaccine [18]. However, the design of such a vaccine is hampered by an incomplete knowledge of which parasite proteins are targets of acquired natural immunity.

The advent of technologies allowing whole genome sequencing at epidemiological scales has allowed investigators to transition from investigation of single antigens to performing genome-wide screens to identify loci likely to be involved in the acquisition of protective immunity to malaria, including uncharacterized genes encoding products of unknown function [21,22]. Although there have been previous genome-wide studies in *P*. *falciparum* to identify immune targets based on genomic signatures of balancing or diversifying selection at a population level [23–27], these studies do not relate identified signatures with clinical outcomes in individuals in endemic areas, making it difficult to directly link such signatures to immune selection. In addition, identification of correlates of protection based strictly on immunological approaches is challenging, as it can be difficult to differentiate responses reflective of exposure *versus* protection [28]. Expanding on our previous approach where we examined the dynamics of vaccine antigen alleles in individuals’ repeated infections over time in relation to the development of symptoms[16,17], we compared whole genome sequence data generated from *P*. *falciparum* infections collected from participants in a longitudinal cohort study conducted in Malawi to identify targets of allele-specific immunity to malaria. Specifically, we compared the frequency of parasite alleles in symptomatic infections occurring in individuals with different levels of malaria immunity to identify significantly differentiated sites, and also compared alleles in repeated infections within an individual *versus* between individuals to identify polymorphic sites that vary most within individuals. Genes identified using both approaches were considered possible immune targets and were further examined for their potential as vaccine candidates. As a proof of concept of the utility of our approach in identifying targets of allele-specific immune responses, we compared the frequency of alleles in one of the identified antigens (previously considered as a potential vaccine candidate) in individuals with different levels of malaria immunity to test the hypothesis that individuals who are more immune become ill when infected with a parasite having rarer alleles to which they have not yet developed immunity.

## Results

### Participant/Infection characteristics and definition of immune status

To identify targets of allele-specific immunity to malaria, we generated whole-genome sequence data from 140 parasite isolates collected from symptomatic infections occurring in participants in a longitudinal cohort study in Malawi [29].

**Fig 1:**
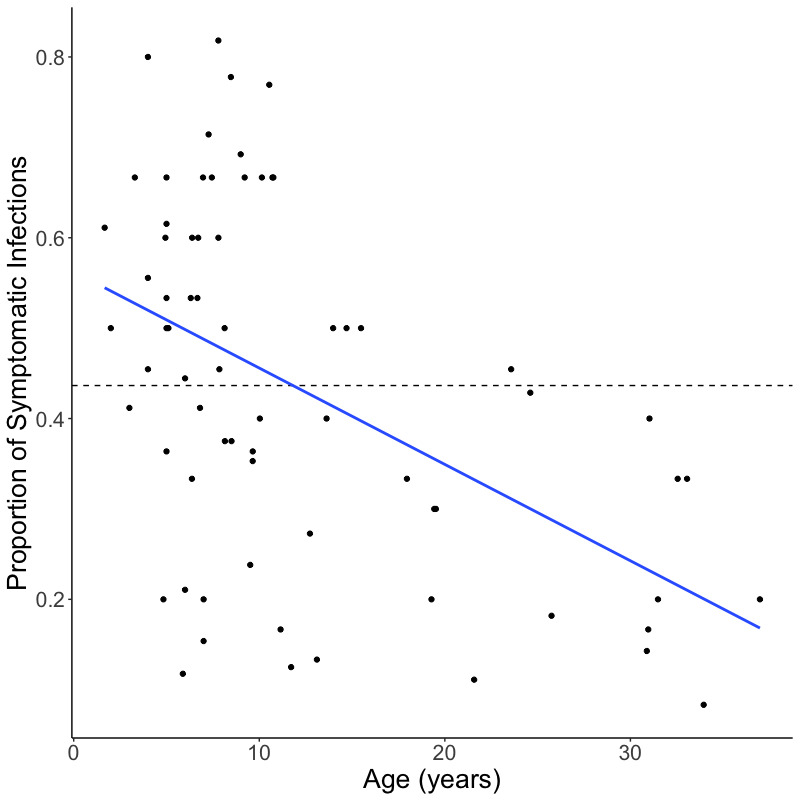
Relationship between proportion of symptomatic infections and age. Scatterplot, including linear regression line (blue), shows the relationship between the proportion of symptomatic infections per individual over the course of the study and age of the individual at enrollment. The dashed line shows median proportion of symptomatic infections, which was used to define the high and low immunity groups.

Although age is often used as a proxy for immune status in high transmission areas [14,30–32], this metric does not account for heterogeneous exposure to infectious mosquito bites, which has been observed in endemic areas [33–38]. To better account for heterogeneous exposure at an individual level, we used the proportion of total infections that were symptomatic over the two-year study period to categorize immune status, using the median as a cutoff to define high and low immunity groups. Although these groups consisted of individuals with a range of ages, the median age (13.2 years) of individuals in the group with high immunity was significantly greater than the median age (7.3 years) of individuals in the low immunity group (*p-*value = 0.0002, Wilcoxon-Mann-Whitney test) (Fig 1 and Table 1), as would be expected in a high malaria transmission setting such as Malawi.

**Table 1:** Participant/infection characteristics in high and low immunity groups^#^.

Characteristics	High Immunity (n = 28)	Low Immunity (n = 33)	*P*-value
Median age in years	13.2 (8.4–27.0)^^^	7.3 (5.0–9.2)	0.0002^+^
Male %	36	52	0.33*
Median parasites/μL	4710 (550–160600)	48200 (26660–171200)	7.74x10^-06^^+^
Median % genome coverage 20x	88.7 (72.8–91.3)	90.1 (79.4–92.0)	0.27^+^
Median depth of coverage	130x (107–170)	156x (112–183]	0.25^+^

^#^High complexity infections lacking a predominant parasite clone were excluded

^^^Interquartile range shown in parentheses

*P-value determined using z-score test for difference in proportions

^+^*P*-value determined using Wilcoxon-Mann-Whitney test

Only one infection from each individual was included in comparisons between the high and low immunity groups, with samples selected in a manner to reduce temporal variability between infections (see Methods). DEploid-IBD [39] was used to estimate the proportion of each parasite clone within an infection. Infections without a predominant clone (i.e., where the majority clone had a frequency <60% within the infection) were defined as complex infections. Although the median frequency of the majority clone was not significantly different between infections in the two immunity groups (S1 Fig, *p-*value = 0.34, Wilcoxon-Mann-Whitney test), the high immunity group had a greater number of complex infections (n = 7) than the low immunity group (n = 2). These nine complex infections were excluded from further analysis to avoid confounding by infection complexity and misclassification of alleles likely contributing to clinical illness.

The median parasite density of infections in the high immunity group was significantly lower than in the low immunity group (Table 1, *p-*value = 7.74 x 10^−06^, Wilcoxon-Mann-Whitney test). However, there was no significant difference in the percentage of the parasite genome with at least 20-fold coverage (Table 1, *p-*value = 0.27, Wilcoxon-Mann-Whitney test), or in the median average depth of coverage (Table 1, *p-*value = 0.25, Wilcoxon-Mann-Whitney test) between whole genome sequence data generated from infections in the two groups.

### Differentiated loci between groups with different levels of immunity to clinical malaria

We hypothesized that, because of allele-specific immune responses, individuals with greater protective immunity to malaria would experience disease when infected with parasite antigen alleles that are rarer in the parasite population, having already developed immunity to more common alleles circulating in the population. Thus, we would expect significant genetic differentiation between antigen alleles in individuals with high *versus* low immunity at loci that are targets of allele-specific immunity.

**Fig 2:**
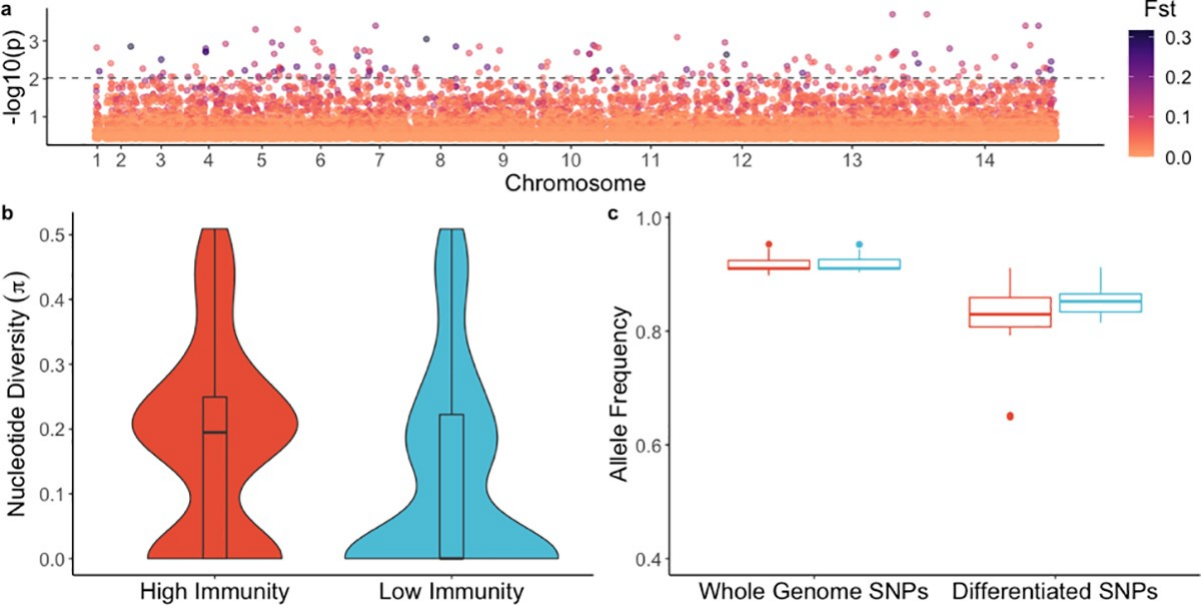
Genetic differentiation between parasites from high immunity *vs*. low immunity groups. a) Genome-wide genetic differentiation (*F*_ST_) between parasites from individuals with higher immunity *vs*. lower immunity. Each point represents a variable, non-synonymous site. Results are plotted as–log_10_ *p*-values on the y-axis. The color of each point represents the *F*_ST_ value, with darker points indicating higher *F*_ST_ values. The dashed line denotes statistical significance (*p-*value = 0.0095), with *p*-value determined by permutation. b) Nucleotide diversity for significantly differentiated SNPs in parasites from individuals with higher immunity and lower immunity. c) Box-plot of mean allele frequency per individual based on SNPs in the whole genome sequences, and which are significantly differentiated SNPs from (a). Red indicates the high immunity group and blue color indicates the low immunity group.

To test this hypothesis, Wright’s fixation index (*F*_ST_), a measure of genetic differentiation between two populations [40], was estimated per non-synonymous single nucleotide polymorphism (SNP) to identify genetically differentiated sites between parasites from the high and low immunity groups with the significance threshold determined through resampling (10,000 permutations). We identified 160 sites (in 145 genes) in the parasite genome that were significantly differentiated between the two immunity groups (*p-*value ≤ 0.009, Fig 2A and S1 Table). Fifty-five of the genes containing significantly differentiated sites (38%) encode proteins of unknown function that are not associated with any computed or curated molecular function or biological process based on Gene Ontology (S1 Table). These 145 gene products included some proteins previously identified as potential vaccine candidates, including AMA1 (apical membrane antigen 1) [17,20], ASP (apical sushi protein, PF3D7_0405900) [41], CLAG8 (cytoadherence linked asexual protein 8, PF3D7_0831600) [42,43], SLARP (sporozoite and liver asparagine-rich protein, PF3D7_1147000) [44] and a conserved protein of unknown function (PF3D7_1359000) [45].

To further test the hypothesis that individuals who are more immune become symptomatic when infected with parasites having antigen alleles that are rarer in the parasite population, we estimated nucleotide diversity at significantly differentiated non-synonymous sites in parasites from the two immunity groups and observed a significantly greater median nucleotide diversity in parasites from the high immunity group compared to parasites from the low immunity group (Fig 2B, *p-*value = 4.74 x 10^−05^, Wilcoxon-Mann-Whitney test). In addition, we estimated the average frequency of alleles in each infection at both significantly differentiated sites and genome-wide variable sites. The median frequency of alleles at genome-wide variable sites was not significantly different between immunity groups (Fig 2C, *p-*value = 0.88, Wilcoxon-Mann-Whitney test); in contrast, at the differentiated sites, the median frequency of alleles was significantly lower in the high immunity group compared to the low immunity group (Fig 2C, *p-*value = 0.007, Wilcoxon-Mann-Whitney test). These results are consistent with the scenario that individuals in the high immunity group are infected with parasites having different lower-frequency alleles compared to individuals in the low immunity group who are infected with parasites sharing more common alleles.

Within 23 polyclonal infections, we also compared the proportion of mismatched alleles between the predominant and minor clones at both significantly differentiated sites and genome-wide variable sites in order to assess whether these clones differ at sites thought to be relevant for immunity. We observed a significantly greater median proportion of mismatches between the major and minor clones within an infection at the differentiated sites compared to genome-wide variable sites (S2 Fig, *p-*value = 8 x 10^−05^, Wilcoxon-Mann-Whitney test), consistent with the hypothesis that the predominant clone represents a breakthrough infection that has escaped allele-specific immune responses that maintain minor clones at a subclinical level.

### Loci that differ more within individuals than between individuals

**Fig 3:**
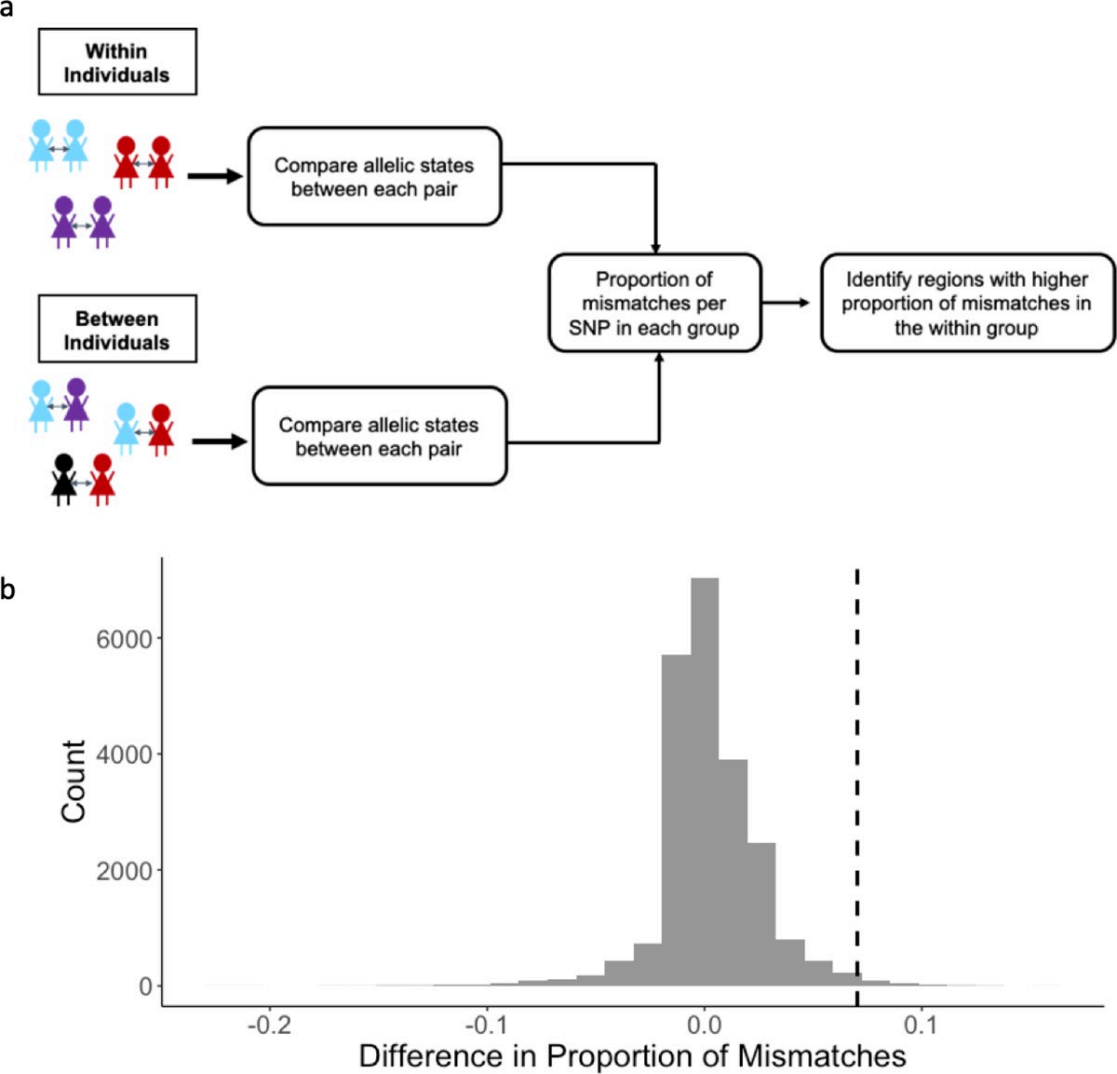
Analysis of mismatches in paired samples within and between individuals. a) Illustration of analysis to identify regions of the genome that vary more in parasites causing illness within the same individual over time (within individuals) compared to random pairs of parasites in the population (between individuals). b) Distribution of differences in the proportion of mismatched alleles in the within group and the between group. The difference was calculated as the proportion of mismatches at each non-synonymous SNP in the within group minus the proportion of mismatches at each non-synonymous SNP in the between group. The dashed black line indicates the threshold for the top 1% most different SNPs in the within group compared to the between group.

We expected that allele-specific immune responses would result in a greater proportion of genetic differences in parasites causing multiple symptomatic infections within an individual compared to parasites causing infection in different individuals, at antigenic loci that are targets of immunity. To identify regions of the parasite genome that are most different in infections occurring within an individual *versus* between individuals, we compared the allelic states at each variable non-synonymous site across the genome between pairs of isolates sampled within an individual and between individuals and estimated the proportion of mismatches per site in each group (Fig 3A). The distribution of the difference in the proportion of mismatches across all non-synonymous variable sites between the two groups (within minus between) is shown in Fig 3B (see also S3A Fig). The difference in the proportion of mismatches at each site had a median and mode equal to zero, indicating that the proportion of mismatches was not different within and between individuals at most sites. There was no significant correlation between the number of days between infections in a pair and the proportion of mismatches (S3B Fig, Pearson’s correlation *r* = 0.065, *p*-value = 0.3), suggesting that time was not a significant confounding factor in the analysis. We further examined the top 1% of sites that differed most within individuals compared to between individuals, which included 223 SNPs, located in 173 genes (S2 Table). Sixty-eight (39%) of these 173 genes encode proteins of unknown function that are not associated with any computed or curated molecular function or biological process based on Gene Ontology, and at least 15 (8.7%) encode for proteins that have been previously identified as potential vaccine candidates.

### Loci identified as possible targets of immunity by both analytical approaches

Twenty-five genes were identified by both analytical approaches, of which 11 (44%) encode proteins of unknown function (Table 2). Based on publicly available data, 20 of the 25 genes have a moderate to high level of expression in the erythrocytic stage of the parasite life cycle [46] (S3 Table). Eight genes have a mutagenesis index score near zero, suggesting that they are likely essential [47], and at least 12 genes have either a known or a predicted transmembrane domain or a signal peptide [48] (S4 Table). Among these 25 gene products are proteins whose putative function make them biologically plausible vaccine candidates, including SURFIN4.2 (thought to be involved in formation of the moving junction during erythrocyte invasion) [49], as well as members of the *clag* and *phist* multigene families (both thought to have a role in parasite cytoadherence) [50,51].

**Table 2:** Gene products identified as likely targets of allele-specific immunity to malaria based on two analytical approaches.

Gene ID	Annotation	GO Function	GO Process
PF3D7_0311900	heptatricopeptide repeat-containing protein, putative	null	null
PF3D7_0312500	major facilitator superfamily-related transporter, putative	null	transmembrane transport
PF3D7_0318200	DNA-directed RNA polymerase II subunit RPB1	DNA-directed 5’-3’ RNA polymerase activity	transcription
PF3D7_0412300	phosphopantothenoylcysteine synthetase, putative	null	null
PF3D7_0421700	conserved Plasmodium protein, unknown function	null	null
PF3D7_0424400	surface-associated interspersed protein 4.2 (SURFIN 4.2)	host cell surface binding	entry into host cell
PF3D7_0511500	RNA pseudouridylate synthase, putative	RNA binding, pseudouridine synthase activity	RNA modification, pseudouridine synthesis
PF3D7_0522400	conserved Plasmodium protein, unknown function	null	protein localization/transport
PF3D7_0526600	conserved Plasmodium protein, unknown function	null	lipid biosynthetic process
PF3D7_0605600	nucleoside diphosphate kinase, putative	nucleoside diphosphate kinase activity	nucleoside diphosphate phosphorylation
PF3D7_0619600	conserved Plasmodium protein, unknown function	null	null
PF3D7_0704600	E3 ubiquitin-protein ligase	ubiquitin-protein transferase activity	response to drug
PF3D7_0710200	conserved Plasmodium protein, unknown function	null	null
PF3D7_0807700	serine protease DegP	serine-type endopeptidase activity	response to oxidative stress and temperature stimulus
PF3D7_0831600	cytoadherence linked asexual protein 8	null	null
PF3D7_0914300	met-10+ like protein, putative	null	null
PF3D7_1004200	WD repeat-containing protein, putative	protein binding	transport
PF3D7_1030400	conserved protein, unknown function	null	null
PF3D7_1033100	S-adenosylmethionine/0rnithine decarboxylase	adenosylmethionine decarboxylase activity	spermidine/spermine biosynthetic processes
PF3D7_1035100	probable protein, unknown function	ATP binding	null
PF3D7_1102500	Plasmodium exported protein (PHISTb), unknown function	null	null
PF3D7_1149600	DnaJ protein, putative	null	null
PF3D7_1219100	clathrin heavy chain, putative	clathrin light chain binding, structural molecule activity	clathrin coat assembly, intracellular protein transport, vesicle-mediated transport
PF3D7_1465800	dynein beta chain, putative	ATP binding, microtubule motor activity	microtubule-based movement
PF3D7_1475900	KELT protein	null	null

**Fig 4:**
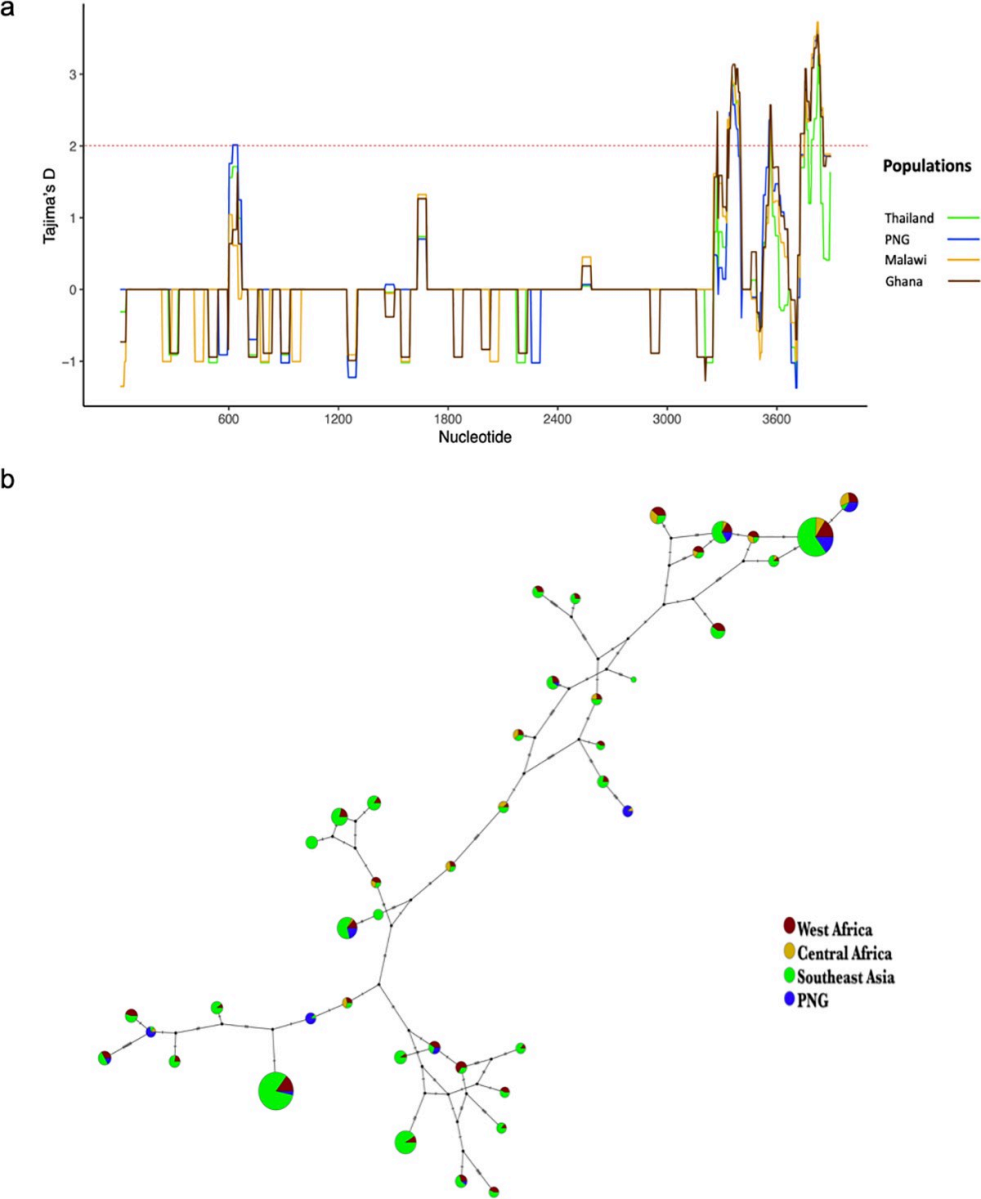
Global diversity of *clag8*. a) Tajima’s D along the *clag8* gene in samples from Thailand, PNG, Malawi, and Ghana. The dotted red lines represent a positive Tajima’s D value (≥ 2), suggestive of balancing selection. b) Haplotype network of *clag8* using *P*. *falciparum* sequence data.

As a proof of concept of the utility of our approach for identifying targets of allele-specific immunity, we used publicly available sequence data from Pf3K [52] combined with 156 sequences from Papua New Guinea (PNG) [53,54] from the MalariaGEN *P*. *falciparum* Community Project [55] to examine global diversity in *clag8*, one of the 25 identified genes that has been considered previously as a potential vaccine candidate, and found high nucleotide diversity, Tajima’s D, and number of segregating sites in the C-terminal region of *clag8*. This pattern was consistent when comparing data from parasites collected in Malawi to data from parasites from other regions of the world (Fig 4A). A haplotype network generated using *clag8* sequences from multiple geographic areas showed no evidence of regional adaptation (Fig 4B), including low *F*_ST_ values between *clag8* sequences from Africa, Asia and PNG (S5 Table). Further analysis of CLAG8 protein sequences show regions with high protein disorder and B-cell epitope sites, especially in the C-terminal region (S4 Fig). To test our initial hypothesis that immune individuals become ill when infected with parasite antigen alleles that are rarer in the parasite population, we compared the frequency of *clag8* haplotypes based on the C-terminal region of the gene in individuals with different levels of immunity and observed that parasites causing illness in individuals in the high immunity group tended to have *clag8* haplotypes that were lower in frequency compared to individuals in the low immunity group, although significance was borderline (S5 Fig, *p*-value = 0.09, Wilcoxon-Mann-Whitney test).

## Discussion

Previous studies have shown evidence of allele-specific acquisition of immunity to *P*. *falciparum* in single genes or proteins [8–10,12,16,17] that have been identified as potential vaccine antigens based on traditional vaccinology approaches that empirically identify immunogenic proteins. Other studies have performed genome-wide screens to identify antigens based on genomic signatures of balancing selection but have not related these signatures to clinical outcomes that might link specific signatures with protective immune responses [23–27]. In this study, we conducted a genome-wide, individual-level analysis to identify targets of allele-specific immunity to clinical malaria using *P*. *falciparum* whole genome sequence data by identifying parasite genes that are genetically differentiated between individuals with different levels of immunity to malaria and genomic regions that are most different in parasites causing illness within the same individual *versus* between individuals. Twenty-five genes were identified using both analytical approaches and encode possible targets of allele-specific acquired immunity to clinical malaria, including genes thought to be involved in erythrocyte invasion and cytoadherence, among other functions. Examination of global diversity in *clag8*, a gene encoding a protein that has previously been considered as a vaccine candidate, provided evidence of immune selection in the C-terminal region of the gene and did not indicate geographical differences that might be indicative of local adaptation or genetic drift. Consistent with the hypothesis of allele-specific acquisition of immunity, *clag8* haplotypes of parasites causing illness in individuals with greater immunity were lower in frequency compared to those causing illness in less immune individuals. These findings support the utility of our approach for identification of targets of allele-specific immunity and further investigation of these 25 loci as potential vaccine candidates.

In this study, we estimated the genetic complexity of infections and observed that individuals in the group with higher immunity generally had more complex infections than individuals with lower immunity. Although not statistically significant, this pattern is broadly consistent with results from other studies that have reported associations between infection complexity and either age and/or risk of disease [16,56]. The greater complexity of infections in individuals with higher immunity is consistent with the idea that immune individuals are capable of maintaining some parasite clones at a subclinical level and could suggest a possible role of polyclonal infections in maintaining immunity through continuous exposure to different strains [57,58]. Alternatively, the difference in complexity of infections between the two groups could also be reflective of transmission levels within the community, rather than just reflective of immunity. In polyclonal infections from our data set, we observed that the predominant clone was more likely to have different alleles than the minor clone at sites identified as potential targets of immunity in our analysis. This finding is consistent with the hypothesis that minority clones are maintained at a subclinical level by acquired immunity, while the predominant clone is able to escape the immune response (because it has unrecognized alleles), resulting in a symptomatic infection. Prior to downstream analysis, infections lacking a predominant clone were excluded to avoid confounding by infection complexity and misclassification of clones likely responsible for disease symptoms. The exclusion of infections lacking a predominant clone led to the removal of a greater proportion of infections from the high immunity group than the low immunity group, which may have resulted in an underestimation of parasite diversity in the high immunity group. However, we do not believe this underestimation impacted our conclusions, as infections within the high immunity group were significantly more diverse at differentiated loci even after exclusion of these highly complex infections.

We hypothesized that individuals who have a higher degree of immunity to malaria would have a greater risk of malaria symptoms when infected with parasites having protein variants that are of lower frequency in the local parasite population, owing to their having already acquired immunity to variants encoded by more common alleles. When we estimated nucleotide diversity at differentiated loci in both immunity groups, we found significantly greater diversity in the group with higher immunity compared to the group with lower immunity. This difference in diversity may reflect the fact that individuals with higher immunity have symptomatic infections with parasite alleles that are rarer in the population and therefore more likely to be different from one another at differentiated loci. This scenario is also supported by our finding that the median frequency of the infecting allele was significantly lower in more immune individuals compared to less immune individuals when examining differentiated loci. These results also demonstrate the importance of accounting for rare alleles in vaccine design, as they may also lead to escape from vaccine-induced immunity.

Genome-wide screens for signatures of balancing selection have previously identified some of the 25 genes that we identified in this study, including PF3D7_0710200, *clag8* and other genes from the *surfin* and *phist* multigene families [23–25,27]. PF3D7_0710200, a gene encoding a conserved protein of unknown function, was identified by four separate studies as a potential immune target [23–25,27]; however, little is known about the function of this protein. Indeed, 11 of the 25 genes (44%) identified in this study encode for proteins of unknown function, highlighting the importance of further genetic screens to determine the role of such genes, which make up ~35% of the parasite genome [59]. A recent study has suggested that most genes involved in host-parasite interactions, including many known antigens, are non-essential [47]; however, eight of the 25 genes identified in this study would be considered essential genes, based on their low mutagenesis index score [47]. Such essential genes may be attractive vaccine targets as there may be less redundancy in function that would allow the parasite to adapt and escape vaccine-induced inhibition.

As a proof of concept of the utility of our approach for identifying targets of allele-specific immunity, we further examined the global diversity of one of the 25 identified genes, *clag8*, which has previously been considered as a malaria vaccine candidate. *clag8* belongs to the *clag* multigene family and is one of the least studied genes in the *clag* family. c*lag8* is highly expressed during the first few hours following erythrocyte invasion (early ring stage), displays decreased expression from 5 hours to 35 hours post invasion, and then increased expression during the schizont stage [46]. It is thought to be part of the RhopH complex, formed by the members of *rhoph1/clag* gene families. The RhopH complex is an erythrocyte-binding protein complex inside the rhoptry and has been suggested to play an important role in establishment of the parasitophorous vacuole [43,60,61]. Previous studies have reported evidence of positive diversifying selection in this gene, with high nucleotide diversity and a high proportion of non-synonymous substitutions per site (*d*_N_) [43]. Our global analysis of *clag8* diversity displayed high nucleotide diversity and Tajima’s D values in the C-terminal region of the gene, as well as evidence of high protein disorder and predicted B-cell epitopes in the C-terminal region of the protein, suggesting that it is likely to be immunogenic [62]. These results were consistent in all the countries included in the dataset, which represented parasite isolates from three major malaria endemic regions [52]. Additionally, *clag8* haplotypes displayed no evidence of geographical adaptation, with major haplotypes being observed in all geographic areas, and seemed to cluster into three main groups. Further studies to identify functional epitopes within the protein and potential cross-reactivity are necessary to determine whether related haplotypes can be grouped into serotypes for the purpose of designing a broadly protective vaccine. At an individual level, and in support of our overarching hypothesis, individuals with higher immunity to malaria were infected with *clag8* haplotypes that were less frequent compared to the *clag8* haplotypes infecting individuals with lower immunity.

It is noteworthy that our combined list of genes based on both analytical approaches did not include leading blood stage vaccine candidates such as AMA1 and the MSPs, although some of these genes were identified in a single approach. Using a protein microarray, Crompton *et al*. found that antibody responses to leading vaccine candidates, such as AMA1, MSP1, and MSP2, did not distinguish individuals who were protected from clinical infection versus those who were not in a cohort of individuals from Mali [15], suggesting the possibility that responses to these proteins are not the primary drivers of clinical immunity. Other studies [17,63,64] have supported the hypothesis that responses to antigens such as AMA1 contribute to allele-specific clinical immunity but may be short-lived. In our analyses, infection pairs within an individual were not necessarily consecutive infections, with the time between infections ranging from 27 to 696 days. Although we did not see a significant correlation between the proportion of allele mismatches and the number of days between infections in a pair, it is possible that we could have failed to identify antigens involved in allele-specific acquisition of immunity that elicit short-lived immune responses. Studies with larger sample size would likely be required to distinguish antigens that have different antibody kinetics.

In addition, analyses in this study included only the core genome, owing to our use of selective whole genome amplification to enrich for parasite DNA prior to sequencing, which has been shown to result in poor sequencing coverage in the telomeric and centromeric regions [65,66]. Limiting analysis to the core genome could have prevented us from identifying members of multigene families that may be important for development of immunity to clinical malaria, as many of these genes are located in these low-coverage regions of the genome. However, because multigene families have been implicated in severe malaria, it is possible that immunity to these diverse antigens may be more relevant to preventing severe rather than uncomplicated malaria.

Other limitations of this study include the inability to examine asymptomatic infections, owing to the difficulty in obtaining whole genome sequence data from these low parasitemia infections, as well as the focus on SNP data *versus* other types of variants, such as indels or structural variants, that could also contribute to allele-specific immunity. Specifically, our approach would not have detected allelic dimorphism observed in repetitive regions of some known malaria vaccine antigens, such as MSP1 and MSP2 [67]. Such low-complexity regions are difficult to resolve using short-read sequencing data as reads spanning these regions do not map uniquely to the reference genome. Targeted sequencing of specific genes with long-read sequencing platforms may allow sequencing of asymptomatic infections and examination of the contribution of additional types of variants to allele-specific immunity.

Here, we describe a promising genome-wide approach to identify potential targets of allele-specific immunity to clinical malaria. This approach identifies individual-level associations between antigen allele dynamics and patient clinical outcomes. Inferences are based on changes in parasite allele frequencies in individuals over time, potentially allowing identification of subdominant, but protective, epitopes that might otherwise be difficult to detect in immunological studies, because they are masked by responses to immunodominant, but not protective, loci. Using this approach, we identified 25 genes, many of unknown function, that encode proteins that can be further characterized for their potential as candidates for a multivalent subunit malaria vaccine through immunological epitope mapping and functional studies of antibodies elicited to these proteins.

## Methods

### Ethics statement

Clinical samples were collected under protocols approved by the ethics committees at the College of Medicine in Blantyre, Malawi, and the University of Maryland, Baltimore. Written informed consent was provided by study participants or their guardians.

### Study design and samples

Parasite isolates were collected from participants in a longitudinal cohort study conducted in the Chikhwawa district of southern Malawi. Details about the participants and study procedures have been described previously by Buchwald *et al* [29]. Briefly, 120 children and adults reporting to the Mfera Health Centre with uncomplicated malaria between June 2014 and March 2015 were followed monthly over two years. Blood samples were collected at each monthly visit and all unscheduled visits where individuals reported to the Health Centre with symptoms of malaria. For each visit, parasitemia was diagnosed by both microscopy and PCR. The data analyzed in this study were generated from red blood cell pellets collected from symptomatic, uncomplicated malaria infections identified during passive follow up. The median parasitemia of the sampled infections as determined by microscopy was 21,960 parasites/μL and ranged from 0 parasites/μL (but positive by a rapid diagnostic test) to 241,260 parasites/μL. All samples were confirmed to be positive for *P*. *falciparum* by PCR. To ensure only independent infections were included in the analysis, infections within an individual separated by <14 days were excluded. DNA from red blood cell pellets was extracted using the method of Zainabadi *et al* [68]. Extracted DNA was enriched for parasite DNA using an optimized selective whole genome amplification approach described by Shah *et al* [65].

### Whole genome sequencing

Genomic DNA libraries were constructed for sequencing using the KAPA Library Preparation Kit (Kapa Biosystems, Woburn, MA). DNA (≥ 200 ng) was fragmented with the Covaris E210 to ~200 bp. Libraries were prepared using a modified version of the manufacturer’s protocol. The DNA was purified between enzymatic reactions and library size selection was performed with AMPure XT beads. Libraries were assessed for concentration and fragment size using the DNA High Sensitivity Assay on the LabChip GX (Perkin Elmer, Waltham, MA). Library concentrations were also assessed by qPCR using the KAPA Library Quantification Kit. Libraries were pooled and subsequently sequenced on an Illumina HiSeq 4000 (Illumina, San Diego, CA) to generate 150 bp paired-end reads.

### Read mapping and SNP calling

Sequencing data were analyzed by mapping raw fastq files to the 3D7 reference genome using Bowtie2 [69]. Binary Alignment Map (BAM) files were processed following the GATK Best Practices workflow to obtain analysis-ready reads [70,71]. Bedtools [72] was used to generate coverage and depth estimates from the processed reads, and the GATK Best Practices workflow was followed for variant calling [70,71]. Haplotype Caller was used to create genomic variant call format (GVCF) files for each sample and joint SNP Calling was performed (GATK v3.7). Variants were removed if they met the following filtering criteria: variant confidence/quality by depth (QD) < 2.0, strand bias (FS) > 60.0, root mean square of the mapping quality (MQ) < 40.0, mapping quality rank sum (MQRankSum) < -12.5, read position rank sum (ReadPosRankSum) < -8.0, quality (QUAL) < 50. Variant sites with >20% missing genotypes and samples with >30% missing data were additionally removed using vcftools. Variants were also removed if the minor allele was not present in at least two samples. Only the core genome was used for further analysis, which has been previously defined by exclusion of the highly variable telomeric and centromeric regions of the genome [73]. The median percentage of the genome covered by ≥ 20 reads was 88.9% [65]. After applying quality-control filters, 55,970 SNPs were called in the core genome, including 22,177 non-synonymous SNPs, with an average 11.6 variants called per gene.

### Definition of immune status

The degree of immunity to clinical malaria was defined based on the proportion of symptomatic infections out of all *P*. *falciparum* infections experienced by each study participant over the course of the two-year study. To account for exposure, individuals with less than five total infections, including symptomatic and asymptomatic infections, were excluded from the analysis. The median proportion of symptomatic infections was used as the cutoff to categorize individuals into higher and lower immunity groups. The limited sample size of our study did not allow us to categorize immune status as an ordinal variable.

### Complexity of infection and genetic differentiation

Only one infection from each individual was included in comparisons between high a low immunity groups. Infections were selected based on proximity to the median of the distribution of sampling dates to reduce temporal variability. DEploid-IBD [39] was used to estimate the proportion of each clone within an infection. Infections without a predominant clone (i.e., where the majority clone had a frequency <60% within the infection) were defined as complex infections and were excluded from downstream analysis. For the remaining samples, the major allele was called at heterozygous positions if the allele was supported by ≥70% of reads; otherwise, the genotype was coded as missing. A Wilcoxon-Mann-Whitney test was used to assess differences in the frequency of the majority clone in infections from the two immunity groups.

Vcftools [74] was used to estimate Weir and Cockerham *F*_ST_ in variable non-synonymous, bi-allelic sites. Significance was determined using 10,000 permutations, where the observed population was resampled without replacement. To determine the impact of performing the analysis based on the predominant allele at biallelic sites, we also performed the analysis using multiallelic sites and all alleles within an infection. Although *F*_ST_ values were generally higher in the analysis with multiple alleles compared to the analysis with a single major allele, sites that were significantly differentiated in the analysis based on the major allele were also significantly differentiated in the analysis where minor alleles were also included. Nucleotide diversity at significantly differentiated sites was estimated using vcftools [74]. PlasmoDB (v44) [22] was used to identify genes containing differentiated SNPs.

In all polyclonal infections, the major and minor clones (defined by clone frequencies obtained from DEploid-IBD [39]) were compared, provided clone frequency was less than 80% and greater than 10% (n = 23). At each non-synonymous site, the proportion of samples with mismatched alleles from major and minor clones was estimated. The proportion of mismatches was then compared between significantly differentiated sites and all remaining variable sites from the genome. The *p*-value was estimated by conducting a Wilcoxon-Mann-Whitney test to determine if there is a significant difference in mismatches between clones at different sites *versus* remaining genome-wide variable sites.

### Paired infection analysis

Individuals with parasite whole genome sequence data from at least two symptomatic infections occurring at least 14 days apart were included in the comparison of infections occurring within the same host to infections occurring in different hosts. Multi-allelic sites were included in the analysis of paired infections, in contrast to analyses of genetic differentiation. The ‘within’ group included all pairs of parasites collected at different time points from the same individual. The ‘between group’, included all pairs of parasites from different individuals. A total of 116 samples were included in this analysis. The within group contained 124 pairs of samples and the between group contained 6546 pairs of samples. For all pairs, the allelic state was compared at each site and the proportion of pairs with non-matching allelic states was estimated by site (illustrated in Fig 3). The difference between the within group and the between group was calculated by subtracting the proportion of pairs with non-matching allelic states for each site. The *p-*value was estimated by conducting a one-sided z-test using the difference in proportion of mismatched alleles between the two groups. PlasmoDB [22] was used to identify genes containing the SNPs of interest.

### Global diversity in *clag8*

The MalariaGEN Pf3K project release 5.1 data [52] was used to estimate global diversity in these genes identified in this study. The Pf3K dataset includes whole genome sequencing data from 2,512 samples collected in multiple locations in Asia and Africa. Data [53,54] from 156 additional isolates from Papua New Guinea were also included in the analysis. VaxPack (https://github.com/BarryLab01/vaxpack) was used for global population genetic analysis. GATKv4.0 was used for variant calling. Samples containing ambiguous bases were removed. Singleton SNPs were converted back to reference to prevent false positive variants. Nucleotide diversity and Tajima’s D were calculated for all polymorphic sites separately for every country that had a sample size greater than 50. Templeton, Crandall, and Sing (TCS) [75] method on PopArt [76] was used to construct the haplotype network using non-synonymous SNPs. Protein disorder region and B-cell epitope regions were predicted using PlasmoSIP [62]. The haplotype frequencies of the C-terminal region in Malawian isolates from different immunity groups were estimated for non-synonymous sites using DnaSP v6 [77].

## Supporting information

S1 Fig Proportion of the infection comprised by the majority clone in infections from the high (n = 35) and low (n = 35) immunity groups. Samples without a predominant clone (≥ 0.6), indicated by the dashed line, were defined as complex infections and were removed from downstream analyses.
(TIF)

S2 Fig Proportion of mismatches between the major and minor clones within 23 polyclonal infections. The median proportion of mismatches was significantly greater at differentiated sites thought to be targets of allele-specific immunity compared to genome-wide sites (*p*-value = 8x10^-05^, Wilcoxon-Mann-Whitney test), consistent with the hypothesis that the predominant clone represents a breakthrough infection that has escaped allele-specific immune responses that maintain minor clones at a subclinical level.
(TIF)

S3 Fig **(a) Proportion of mismatched alleles within individuals *vs*. between individuals.** Each point is the proportion of mismatches at a non-synonymous SNP. Black points represent the top 1% most mismatched alleles within individuals. **(b) Correlation between proportion of mismatched SNPs per pair within individuals (y-axis) and time between infections (x-axis).** The blue line represents the linear regression line with 95% confidence region shown by the shaded region.
(TIF)

S4 Fig Predicted protein disorder and B-cell epitopes in *clag8*. The orange line and blocks show the linear B-cell epitope mapping score and predicted B-cell epitope sites, respectively. The blue line and blocks show the protein disorder score and highly disordered region, respectively. The asterisks along the bottom represent known SNPs.
(TIF)

S5 Fig Haplotype frequency of *clag8* c-terminal region (amino acid position > 1000), from Malawian whole-genome sequences used in this study, in individuals from both the immunity groups. (TIF)

S1 Table Significantly differentiated SNPs in parasites infecting individuals with different levels of immunity to clinical malaria. (DOCX)

S2 Table Top 1% most different SNPs within individuals compared to between individuals. (DOCX)

S3 Table Heatmap of Fragments Per Kilobase of transcript per Million mapped reads (FPKM) values to show expression of 25 identified genes during the intraerythrocytic stages of the parasite life-cycle. (DOCX)

S4 Table Protein/Gene features for genes identified with both analytical approaches. (DOCX)

S5 Table Pairwise genetic differentiation (*F_ST_*) between *clag8* sequences from Africa, Asia and Papua New Guinea (PNG). (DOCX)

S6 Table NCBI accession numbers. (DOCX)
