# Supplementary figures and images for: Whole-genome analysis of Malawian Plasmodium falciparum isolates identifies possible targets of allele-specific immunity to clinical malaria
Source: PLoS Genet. 2021 May 25;17(5):e1009576. doi: 10.1371/journal.pgen.1009576 (PMC8184011; doi:10.1371/journal.pgen.1009576)

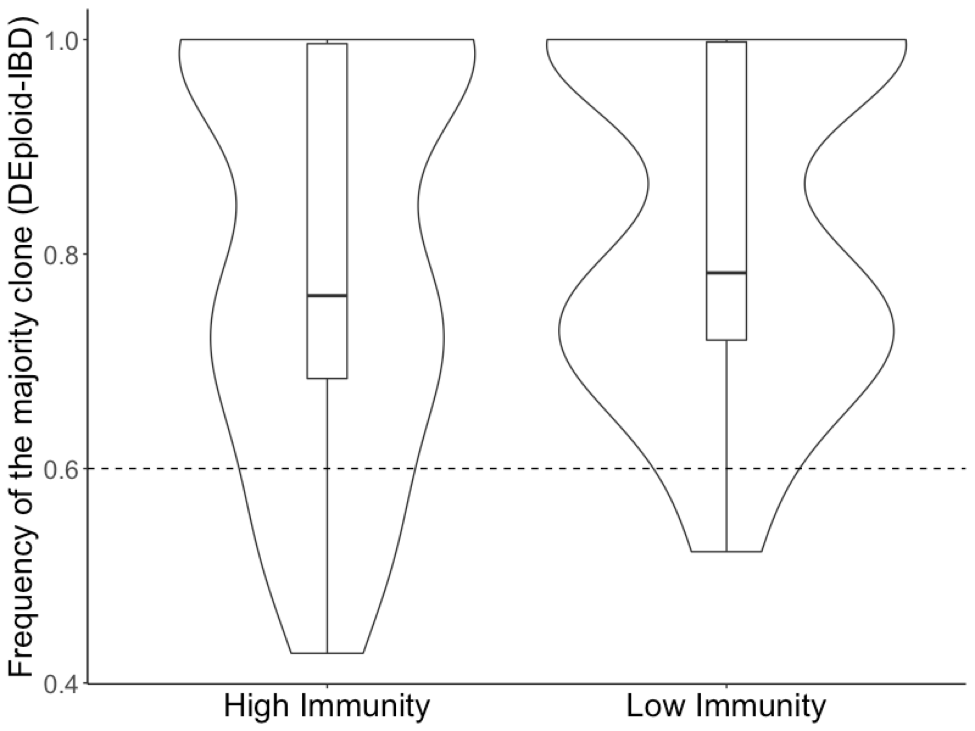

Supplement: S1 Fig — Samples without a predominant clone (≥ 0.6), indicated by the dashed line, were defined as complex infections and were removed from downstream analyses. (TIF) [file pgen.1009576.s001.tif]

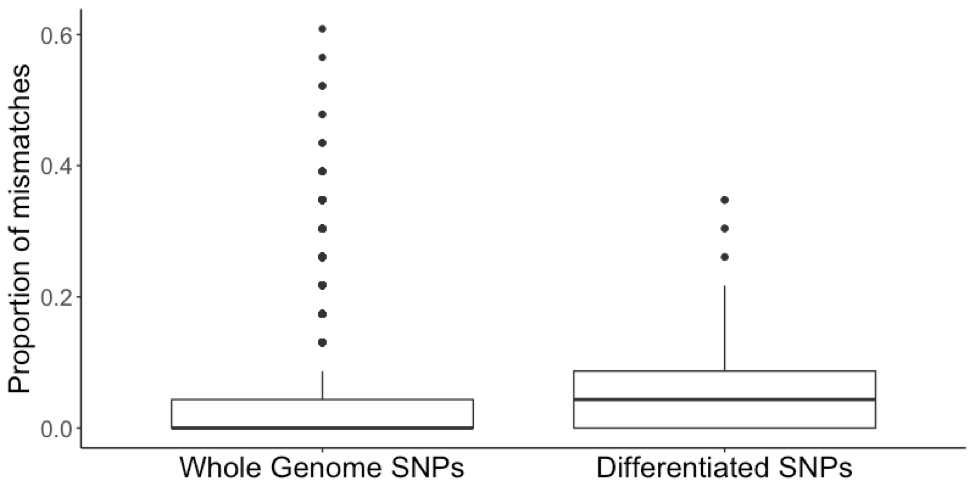

Supplement: S2 Fig — The median proportion of mismatches was significantly greater at differentiated sites thought to be targets of allele-specific immunity compared to genome-wide sites (p-value = 8x10^-05^, Wilcoxon-Mann-Whitney test), consistent with the hypothesis that the predominant clone represents a breakthrough infection that has escaped allele-specific immune responses that maintain minor clones at a subclinical level. (TIF) [file pgen.1009576.s002.tif]

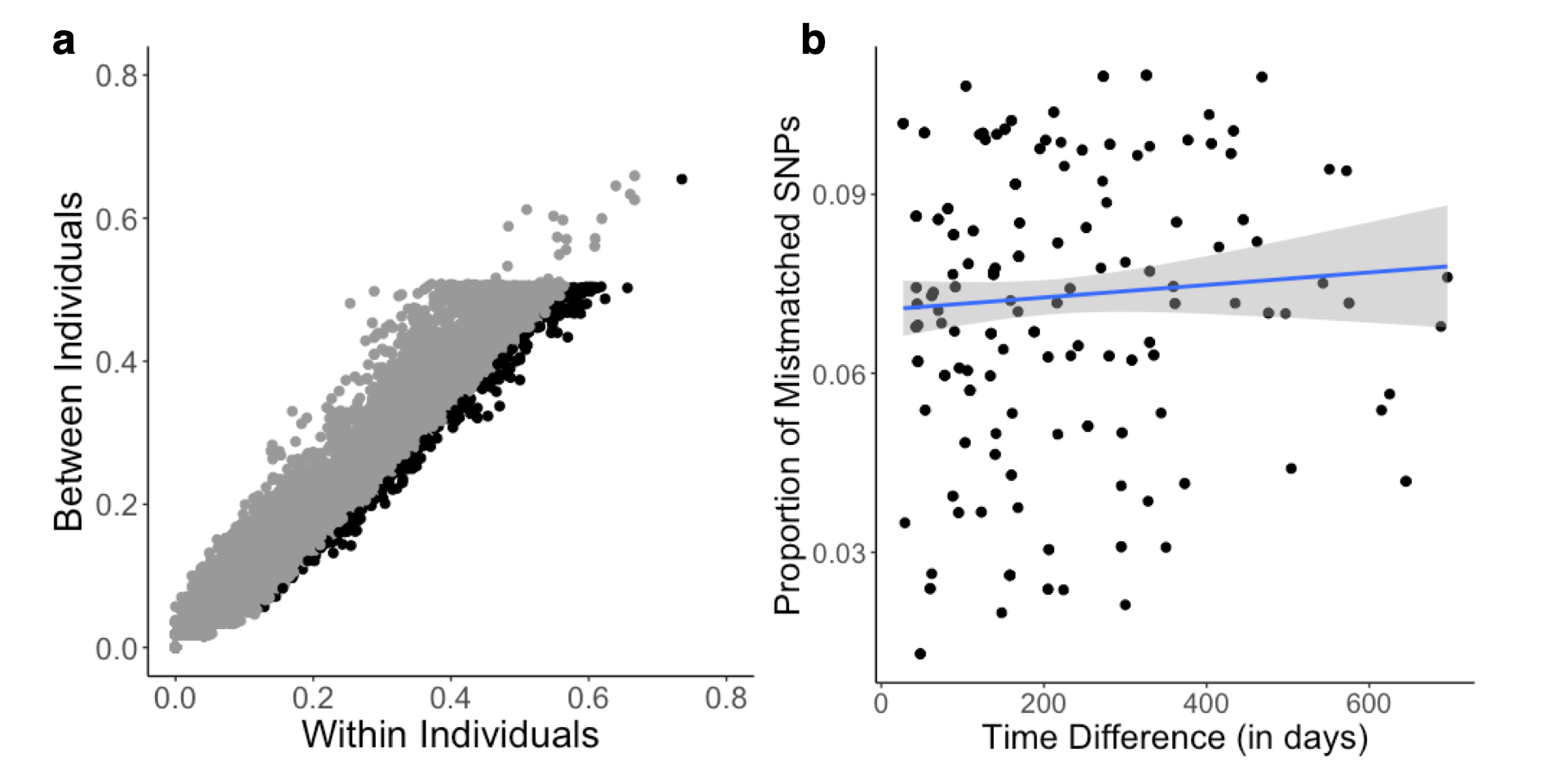

Supplement: S3 Fig — (a) Proportion of mismatched alleles within individuals vs. between individuals. Each point is the proportion of mismatches at a non-synonymous SNP. Black points represent the top 1% most mismatched alleles within individuals. (b) Correlation between proportion of mismatched SNPs per pair within individuals (y-axis) and time between infections (x-axis). The blue line represents the linear regression line with 95% confidence region shown by the shaded region. (TIF) [file pgen.1009576.s003.tif]

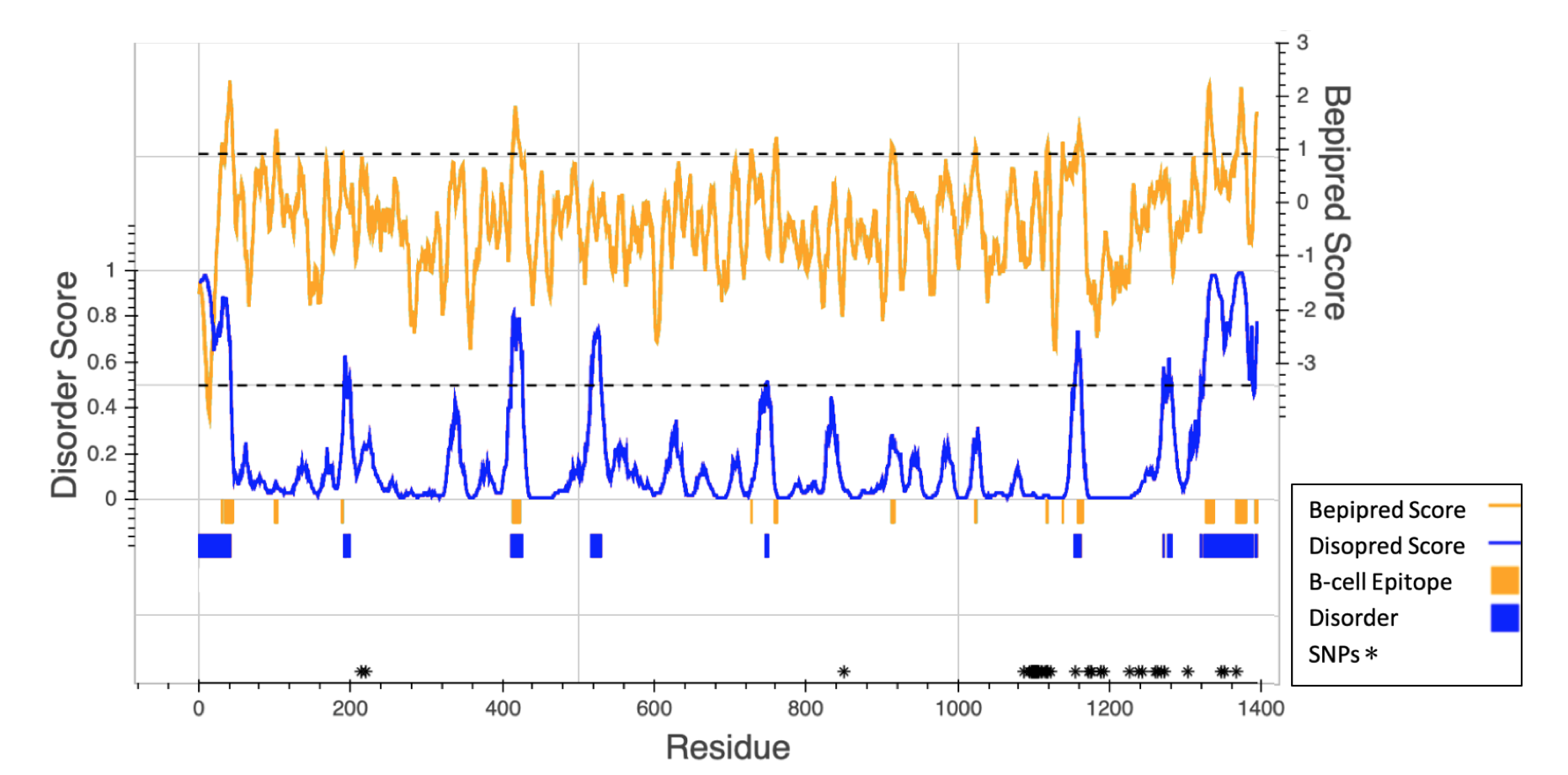

Supplement: S4 Fig — The orange line and blocks show the linear B-cell epitope mapping score and predicted B-cell epitope sites, respectively. The blue line and blocks show the protein disorder score and highly disordered region, respectively. The asterisks along the bottom represent known SNPs. (TIF) [file pgen.1009576.s004.tif]

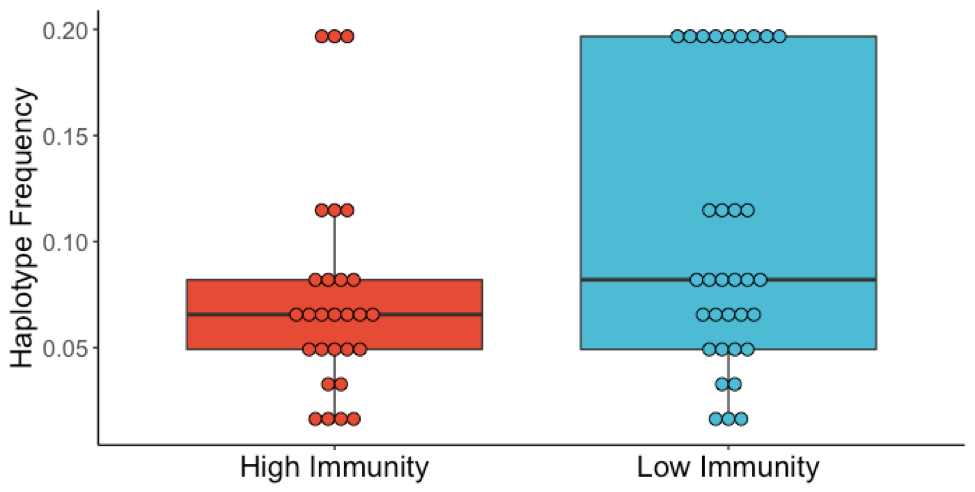

Supplement: S5 Fig — (TIF) [file pgen.1009576.s005.tif]
