# Supplementary material for: Whole-genome analysis of Malawian Plasmodium falciparum isolates identifies possible targets of allele-specific immunity to clinical malaria
Source: PLoS Genet. 2021 May 25;17(5):e1009576. doi: 10.1371/journal.pgen.1009576 (PMC8184011; doi:10.1371/journal.pgen.1009576)
Supplement: S1 Table — (DOCX) [file pgen.1009576.s006.docx]

**S1 Table**. **Significantly differentiated SNPs in parasites infecting individuals with different levels of immunity to malaria.**

| **Gene ID** | **Position** | ***F*_ST_** | ***P*-value** | **Annotation** |
| --- | --- | --- | --- | --- |
| PF3D7_0113300 | 506143 | 0.0832 | 0.0015 | Plasmodium exported protein (hyp1), unknown function |
| PF3D7_0114700 | 563801 | 0.2358 | 0.0063 | rifin |
| PF3D7_0206300 | 254378 | 0.0568 | 0.0039 | pentafunctional AROM polypeptide, putative, pseudogene |
| PF3D7_0206300 | 257473 | 0.0891 | 0.0088 | pentafunctional AROM polypeptide, putative, pseudogene |
| PF3D7_0217000 | 705904 | 0.3158 | 0.0014 | conserved Plasmodium membrane protein, unknown function |
| PF3D7_0302100 | 115739 | 0.0832 | 0.0054 | serine/threonine protein kinase |
| PF3D7_0307700 | 332302 | 0.1560 | 0.0092 | conserved Plasmodium protein, unknown function |
| PF3D7_0311900 | 511434 | 0.2169 | 0.0089 | heptatricopeptide repeat-containing protein, putative |
| PF3D7_0312500 | 530553 | 0.2756 | 0.0031 | major facilitator superfamily-related transporter, putative |
| PF3D7_0314200 | 573820 | 0.1248 | 0.0070 | conserved Plasmodium protein, unknown function |
| PF3D7_0318200 | 752038 | 0.0606 | 0.0087 | DNA-directed RNA polymerase II subunit RPB1 |
| PF3D7_0319400 | 811855 | 0.0534 | 0.0089 | kinesin-8, putative |
| PF3D7_0402300 | 144434 | 0.1463 | 0.0090 | reticulocyte binding protein homologue 1 |
| PF3D7_0405900 | 317120 | 0.0497 | 0.0047 | apical sushi protein |
| PF3D7_0406800 | 352802 | 0.0832 | 0.0069 | ribosomal protein L25, putative |
| PF3D7_0412300 | 544564 | 0.2289 | 0.0016 | phosphopantothenoylcysteine synthetase, putative |
| PF3D7_0412300 | 544537 | 0.2634 | 0.0019 | phosphopantothenoylcysteine synthetase, putative |
| PF3D7_0412300 | 544534 | 0.2634 | 0.0020 | phosphopantothenoylcysteine synthetase, putative |
| PF3D7_0421700 | 992686 | 0.1195 | 0.0012 | conserved Plasmodium protein, unknown function |
| PF3D7_0424400 | 1100345 | 0.1260 | 0.0070 | surface-associated interspersed protein 4.2 (SURFIN 4.2) |
| PF3D7_0424600 | 1113469 | 0.0534 | 0.0075 | Plasmodium exported protein (PHISTb), unknown function |
| PF3D7_0506900 | 288857 | 0.2160 | 0.0060 | rhomboid protease ROM4 |
| PF3D7_0511500 | 510223 | 0.1221 | 0.0041 | RNA pseudouridylate synthase, putative |
| PF3D7_0512200 | 530193 | 0.1248 | 0.0005 | glutathione synthetase |
| PF3D7_0514300 | 600095 | 0.1618 | 0.0086 | aspartate--tRNA ligase, putative |
| PF3D7_0520100 | 832903 | 0.1250 | 0.0088 | protein phosphatase PPM9, putative |
| PF3D7_0520700 | 848187 | 0.0534 | 0.0078 | CDC73 domain-containing protein, putative |
| PF3D7_0522400 | 910775 | 0.1608 | 0.0011 | conserved Plasmodium protein, unknown function |
| PF3D7_0522900 | 951120 | 0.1184 | 0.0022 | zinc finger protein, putative |
| PF3D7_0522900 | 949158 | 0.2078 | 0.0065 | zinc finger protein, putative |
| PF3D7_0525200 | 1049515 | 0.0983 | 0.0061 | structural maintenance of chromosomes protein 6, putative |
| PF3D7_0525900 | 1075441 | 0.0568 | 0.0049 | NIMA related kinase 2 |
| PF3D7_0526000 | 1076999 | 0.0647 | 0.0083 | RAP protein, putative |
| PF3D7_0526100 | 1079955 | 0.0854 | 0.0053 | conserved Plasmodium membrane protein, unknown function |
| PF3D7_0526600 | 1114485 | 0.1577 | 0.0013 | conserved Plasmodium protein, unknown function |
| PF3D7_0526700 | 1119264 | 0.2215 | 0.0056 | conserved protein, unknown function |
| PF3D7_0531400 | 1283805 | 0.0914 | 0.0085 | conserved Plasmodium protein, unknown function |
| PF3D7_0603600 | 145570 | 0.2359 | 0.0048 | conserved Plasmodium protein, unknown function |
| PF3D7_0603900 | 162192 | 0.0914 | 0.0005 | conserved Plasmodium protein, unknown function |
| PF3D7_0604500 | 193129 | 0.0534 | 0.0087 | conserved Plasmodium protein, unknown function |
| PF3D7_0605600 | 232071 | 0.0479 | 0.0094 | nucleoside diphosphate kinase, putative |
| PF3D7_0607600 | 315735 | 0.1458 | 0.0093 | spindle assembly abnormal protein 6, putative |
| PF3D7_0612200 | 516450 | 0.0957 | 0.0011 | leucine-rich repeat protein |
| PF3D7_0613900 | 580541 | 0.0832 | 0.0017 | myosin E, putative |
| PF3D7_0615200 | 626222 | 0.1195 | 0.0046 | conserved protein, unknown function |
| PF3D7_0615400 | 638394 | 0.1124 | 0.0089 | ribonuclease, putative |
| PF3D7_0615900 | 664346 | 0.0539 | 0.0020 | protein phosphatase, putative |
| PF3D7_0619600 | 825760 | 0.2067 | 0.0092 | conserved Plasmodium protein, unknown function |
| PF3D7_0623100 | 943311 | 0.2185 | 0.0048 | nuclear polyadenylated RNA-binding protein NAB2, putative |
| PF3D7_0623700 | 962226 | 0.0876 | 0.0059 | ATP-dependent RNA helicase SUV3, putative |
| PF3D7_0623700 | 962229 | 0.0606 | 0.0081 | ATP-dependent RNA helicase SUV3, putative |
| PF3D7_0703400 | 131434 | 0.0832 | 0.0062 | conserved Plasmodium protein, unknown function |
| PF3D7_0704300 | 198673 | 0.0854 | 0.0017 | conserved Plasmodium membrane protein, unknown function |
| PF3D7_0704600 | 223938 | 0.2617 | 0.0025 | E3 ubiquitin-protein ligase |
| PF3D7_0704700 | 234670 | 0.0539 | 0.0093 | phosphopantetheine adenylyltransferase, putative |
| PF3D7_0708200 | 374723 | 0.1300 | 0.0018 | conserved Plasmodium protein, unknown function |
| PF3D7_0710200 | 471403 | 0.1221 | 0.0037 | conserved Plasmodium protein, unknown function |
| PF3D7_0710200 | 466576 | 0.1923 | 0.0083 | conserved Plasmodium protein, unknown function |
| PF3D7_0710900 | 485072 | 0.0832 | 0.0060 | 50S ribosomal protein L1, mitochondrial, putative |
| PF3D7_0711100 | 492725 | 0.0854 | 0.0054 | conserved protein, unknown function |
| PF3D7_0711200 | 496438 | 0.0854 | 0.0019 | conserved Plasmodium protein, unknown function |
| PF3D7_0711200 | 496337 | 0.2020 | 0.0064 | conserved Plasmodium protein, unknown function |
| PF3D7_0713900 | 631997 | 0.1573 | 0.0004 | conserved Plasmodium protein, unknown function |
| PF3D7_0716700 | 730056 | 0.0891 | 0.0079 | conserved Plasmodium protein, unknown function |
| PF3D7_0717100 | 742201 | 0.0902 | 0.0088 | conserved Plasmodium protein, unknown function |
| PF3D7_0719400 | 853259 | 0.2418 | 0.0053 | conserved Plasmodium protein, unknown function |
| PF3D7_0724000 | 1009402 | 0.0534 | 0.0062 | Rab GTPase activator and protein kinase, putative |
| PF3D7_0801900 | 141983 | 0.0832 | 0.0060 | lysine-specific histone demethylase, putative |
| PF3D7_0802100 | 158232 | 0.1221 | 0.0051 | AP2 domain transcription factor, putative |
| PF3D7_0803200 | 205800 | 0.0810 | 0.0076 | filament assembling protein, putative |
| PF3D7_0807700 | 398541 | 0.3133 | 0.0009 | serine protease DegP |
| PF3D7_0824400 | 1062654 | 0.2479 | 0.0014 | nucleoside transporter 2 |
| PF3D7_0831600 | 1362926 | 0.0810 | 0.0021 | cytoadherence linked asexual protein 8 |
| PF3D7_0904900 | 230882 | 0.0832 | 0.0014 | copper-transporting ATPase |
| PF3D7_0904900 | 228219 | 0.0534 | 0.0068 | copper-transporting ATPase |
| PF3D7_0908900 | 407829 | 0.2309 | 0.0049 | conserved Plasmodium protein, unknown function |
| PF3D7_0914300 | 615470 | 0.0568 | 0.0054 | met-10+ like protein, putative |
| PF3D7_0916400 | 685239 | 0.0854 | 0.0018 | conserved Plasmodium protein, unknown function |
| PF3D7_1003400 | 157280 | 0.1641 | 0.0047 | conserved Plasmodium protein, unknown function |
| PF3D7_1004200 | 190637 | 0.0479 | 0.0094 | WD repeat-containing protein, putative |
| PF3D7_1006400 | 267973 | 0.0568 | 0.0048 | heptatricopeptide repeat-containing protein, putative |
| PF3D7_1007300 | 295733 | 0.0568 | 0.0036 | RING zinc finger protein, putative |
| PF3D7_1022800 | 957933 | 0.1328 | 0.0020 | 4-hydroxy-3-methylbut-2-en-1-yl diphosphate synthase (ferredoxin) |
| PF3D7_1030400 | 1242160 | 0.1432 | 0.0022 | conserved protein, unknown function |
| PF3D7_1030400 | 1242165 | 0.1432 | 0.0022 | conserved protein, unknown function |
| PF3D7_1032900 | 1320068 | 0.0902 | 0.0095 | RNA polymerase II-associated protein 1, putative |
| PF3D7_1032900 | 1320086 | 0.0902 | 0.0095 | RNA polymerase II-associated protein 1, putative |
| PF3D7_1033100 | 1327326 | 0.1780 | 0.0013 | S-adenosylmethionine decarboxylase/ornithine decarboxylase |
| PF3D7_1033200 | 1336216 | 0.1532 | 0.0092 | early transcribed membrane protein 10.2 |
| PF3D7_1033200 | 1336213 | 0.1532 | 0.0095 | early transcribed membrane protein 10.2 |
| PF3D7_1033600 | 1346014 | 0.0832 | 0.0067 | pre-mRNA-splicing factor CEF1, putative |
| PF3D7_1034500 | 1373504 | 0.0832 | 0.0014 | conserved Plasmodium protein, unknown function |
| PF3D7_1035000 | 1387595 | 0.0832 | 0.0060 | U2 snRNA/tRNA pseudouridine synthase, putative |
| PF3D7_1035100 | 1392510 | 0.2419 | 0.0065 | probable protein, unknown function |
| PF3D7_1036700 | 1447194 | 0.0854 | 0.0015 | phosducin-like protein 2, putative |
| PF3D7_1102500 | 119609 | 0.2354 | 0.0060 | Plasmodium exported protein (PHISTb), unknown function |
| PF3D7_1110900 | 432473 | 0.0497 | 0.0052 | ES2 protein, putative |
| PF3D7_1113000 | 493010 | 0.0832 | 0.0065 | conserved Plasmodium protein, unknown function |
| PF3D7_1116000 | 606391 | 0.0876 | 0.0085 | rhoptry neck protein 4 |
| PF3D7_1129300 | 1133484 | 0.0902 | 0.0093 | conserved Plasmodium protein, unknown function |
| PF3D7_1133400 | 1294711 | 0.0891 | 0.0077 | apical membrane antigen 1 |
| PF3D7_1141100 | 1650593 | 0.0891 | 0.0008 | conserved Plasmodium protein, unknown function |
| PF3D7_1147000 | 1861545 | 0.2094 | 0.0057 | sporozoite and liver stage asparagine-rich protein |
| PF3D7_1149600 | 2001090 | 0.1221 | 0.0074 | DnaJ protein, putative |
| PF3D7_1204100 | 201573 | 0.0891 | 0.0072 | conserved Plasmodium protein, unknown function |
| PF3D7_1208400 | 394228 | 0.1386 | 0.0082 | amino acid transporter, putative |
| PF3D7_1208800 | 406174 | 0.0854 | 0.0045 | zinc finger protein, putative |
| PF3D7_1217200 | 679789 | 0.0891 | 0.0037 | multiple RNA-binding domain-containing protein 1, putative |
| PF3D7_1217900 | 701606 | 0.0854 | 0.0052 | PPPDE peptidase domain-containing protein, putative |
| PF3D7_1218400 | 722167 | 0.1221 | 0.0011 | triose or hexose phosphate/phosphate translocator, putative |
| PF3D7_1219100 | 760531 | 0.3170 | 0.0023 | clathrin heavy chain, putative |
| PF3D7_1219100 | 762925 | 0.0832 | 0.0058 | clathrin heavy chain, putative |
| PF3D7_1228800 | 1170044 | 0.1328 | 0.0042 | WD repeat-containing protein, putative |
| PF3D7_1231400 | 1292091 | 0.0891 | 0.0083 | amino acid transporter, putative |
| PF3D7_1235900 | 1500893 | 0.0927 | 0.0092 | pre-mRNA-splicing factor SYF1, putative |
| PF3D7_1245200 | 1889129 | 0.0563 | 0.0081 | conserved Plasmodium membrane protein, unknown function |
| PF3D7_1252200 | 2126271 | 0.1577 | 0.0046 | chitinase |
| PF3D7_1252400 | 2140511 | 0.0854 | 0.0057 | reticulocyte binding protein homologue 3, pseudogene |
| PF3D7_1302700 | 145577 | 0.0876 | 0.0031 | ATP-dependent RNA helicase DHR1, putative |
| PF3D7_1306600 | 315401 | 0.2270 | 0.0070 | V-type proton ATPase subunit H, putative |
| PF3D7_1309300 | 429103 | 0.1248 | 0.0078 | U4/U6 small nuclear ribonucleoprotein PRP3, putative |
| PF3D7_1309700 | 442783 | 0.0832 | 0.0027 | vacuolar protein sorting-associated protein 18, putative |
| PF3D7_1312100 | 513556 | 0.0568 | 0.0037 | GYF domain-containing protein, putative |
| PF3D7_1313600 | 580618 | 0.0568 | 0.0036 | conserved protein, unknown function |
| PF3D7_1321500 | 893641 | 0.0927 | 0.0049 | 3',5'-cyclic nucleotide phosphodiesterase beta, unspecified product |
| PF3D7_1321500 | 893641 | 0.0927 | 0.0049 | 3',5'-cyclic nucleotide phosphodiesterase beta, unspecified product |
| PF3D7_1323800 | 988389 | 0.0854 | 0.0064 | vacuolar protein sorting-associated protein 52, putative |
| PF3D7_1333200 | 1364294 | 0.0568 | 0.0054 | ubiquitin-activating enzyme |
| PF3D7_1340500 | 1615644 | 0.1184 | 0.0071 | conserved Plasmodium protein, unknown function |
| PF3D7_1343800 | 1744884 | 0.0832 | 0.0065 | conserved Plasmodium protein, unknown function |
| PF3D7_1344100 | 1765540 | 0.0832 | 0.0058 | TLD domain-containing protein, putative |
| PF3D7_1347700 | 1910058 | 0.1300 | 0.0056 | ethanolamine-phosphate cytidylyltransferase |
| PF3D7_1350900 | 2028373 | 0.0606 | 0.0028 | AP2 domain transcription factor AP2-O4, putative |
| PF3D7_1359000 | 2341077 | 0.0832 | 0.0024 | conserved Plasmodium protein, unknown function |
| PF3D7_1359100 | 2348407 | 0.1416 | 0.0000 | riboflavin kinase, putative |
| PF3D7_1359600 | 2372539 | 0.0832 | 0.0021 | conserved Plasmodium protein, unknown function |
| PF3D7_1359700 | 2391868 | 0.0832 | 0.0022 | conserved Plasmodium protein, unknown function |
| PF3D7_1360700 | 2426432 | 0.1494 | 0.0019 | E3 SUMO-protein ligase PIAS, putative |
| PF3D7_1364400 | 2587409 | 0.0854 | 0.0048 | conserved Plasmodium protein, unknown function |
| PF3D7_1364400 | 2591509 | 0.0993 | 0.0087 | conserved Plasmodium protein, unknown function |
| PF3D7_1401700 | 66304 | 0.1814 | 0.0022 | conserved Plasmodium protein, unknown function |
| PF3D7_1403800 | 138977 | 0.2537 | 0.0039 | nuclear formin-like protein MISFIT, putative |
| PF3D7_1408700 | 335155 | 0.1248 | 0.0002 | conserved protein, unknown function |
| PF3D7_1411400 | 465740 | 0.0568 | 0.0056 | plastid replication-repair enzyme |
| PF3D7_1420500 | 851461 | 0.1639 | 0.0016 | protein GPR89, putative |
| PF3D7_1427000 | 1053925 | 0.0876 | 0.0086 | conserved Plasmodium protein, unknown function |
| PF3D7_1428600 | 1127489 | 0.1221 | 0.0040 | peptide chain release factor 1 |
| PF3D7_1429400 | 1158319 | 0.0832 | 0.0062 | rRNA (adenosine-2'-O-)-methyltransferase, putative |
| PF3D7_1436200 | 1474255 | 0.0854 | 0.0051 | conserved Plasmodium protein, unknown function |
| PF3D7_1437400 | 1518059 | 0.0606 | 0.0089 | pantothenate kinase 2, putative |
| PF3D7_1445600 | 1873117 | 0.0914 | 0.0027 | RNA-binding protein, putative |
| PF3D7_1456400 | 2310058 | 0.1706 | 0.0045 | conserved Plasmodium protein, unknown function |
| PF3D7_1462900 | 2562559 | 0.1248 | 0.0004 | conserved Plasmodium protein, unknown function |
| PF3D7_1465800 | 2667660 | 0.0854 | 0.0013 | dynein beta chain, putative |
| PF3D7_1469600 | 2856227 | 0.1608 | 0.0004 | acetyl-CoA carboxylase |
| PF3D7_1469600 | 2860791 | 0.1947 | 0.0067 | acetyl-CoA carboxylase |
| PF3D7_1473200 | 2982136 | 0.0568 | 0.0051 | DnaJ protein, putative |
| PF3D7_1474300 | 3041228 | 0.1248 | 0.0053 | DNA repair metallo-beta-lactamase protein, putative |
| PF3D7_1475900 | 3125749 | 0.2393 | 0.0058 | KELT protein |
| PF3D7_1476300 | 3140314 | 0.2334 | 0.0035 | Plasmodium exported protein (PHISTb), unknown function |
