# Supplementary material for: Whole-genome analysis of Malawian Plasmodium falciparum isolates identifies possible targets of allele-specific immunity to clinical malaria
Source: PLoS Genet. 2021 May 25;17(5):e1009576. doi: 10.1371/journal.pgen.1009576 (PMC8184011; doi:10.1371/journal.pgen.1009576)
Supplement: S2 Table — (DOCX) [file pgen.1009576.s007.docx]

**S2 Table. Top 1% most different SNPs within individuals compared to between individuals.**

|  |  | **Proportion of Mismatches** | | |  |
| --- | --- | --- | --- | --- | --- |
| **GeneID** | **Position** | **Between** | **Within** | **Difference*** | **Annotation** |
| PF3D7_0113800 | 528425 | 0.1208 | 0.2018 | 0.081 | DBL containing protein, unknown function |
| PF3D7_0113800 | 531963 | 0.369 | 0.4557 | 0.0867 | DBL containing protein, unknown function |
| PF3D7_0113800 | 533072 | 0.1802 | 0.2688 | 0.0887 | DBL containing protein, unknown function |
| PF3D7_0113800 | 534181 | 0.5031 | 0.5915 | 0.0885 | DBL containing protein, unknown function |
| PF3D7_0113800 | 534236 | 0.5025 | 0.6563 | 0.1538 | DBL containing protein, unknown function |
| PF3D7_0113800 | 534856 | 0.4482 | 0.5294 | 0.0812 | DBL containing protein, unknown function |
| PF3D7_0207000 | 278306 | 0.0568 | 0.1287 | 0.0719 | merozoite surface protein 4 |
| PF3D7_0207800 | 315716 | 0.2461 | 0.3176 | 0.0715 | serine repeat antigen 3 |
| PF3D7_0209000 | 373891 | 0.3984 | 0.4783 | 0.0799 | 6-cysteine protein P230 |
| PF3D7_0209000 | 373912 | 0.5018 | 0.5823 | 0.0805 | 6-cysteine protein P231 |
| PF3D7_0209000 | 374062 | 0.1667 | 0.2525 | 0.0858 | 6-cysteine protein P232 |
| PF3D7_0209000 | 374197 | 0.4796 | 0.5915 | 0.112 | 6-cysteine protein P233 |
| PF3D7_0209400 | 389315 | 0.1621 | 0.25 | 0.0879 | conserved protein, unknown function |
| PF3D7_0212500 | 517408 | 0.2535 | 0.35 | 0.0965 | conserved Plasmodium protein, unknown function |
| PF3D7_0216500 | 680917 | 0.2327 | 0.3097 | 0.0771 | conserved Plasmodium protein, unknown function |
| PF3D7_0219700 | 784786 | 0.5037 | 0.6087 | 0.105 | Plasmodium exported protein (PHISTc), unknown function |
| PF3D7_0220800 | 839251 | 0.5033 | 0.5778 | 0.0745 | cytoadherence linked asexual protein 2 |
| PF3D7_0220800 | 843078 | 0.1996 | 0.2788 | 0.0793 | cytoadherence linked asexual protein 3 |
| PF3D7_0305100 | 246032 | 0.1817 | 0.2617 | 0.08 | conserved Plasmodium protein, unknown function |
| PF3D7_0311900 | 510000 | 0.494 | 0.5842 | 0.0902 | heptatricopeptide repeat-containing protein, putative |
| PF3D7_0312500 | 530553 | 0.4599 | 0.5443 | 0.0844 | major facilitator superfamily-related transporter, putative |
| PF3D7_0315600 | 637453 | 0.2697 | 0.3409 | 0.0712 | zinc finger protein, putative |
| PF3D7_0316200 | 654095 | 0.4752 | 0.5714 | 0.0963 | conserved Plasmodium protein, unknown function |
| PF3D7_0318200 | 751991 | 0.4949 | 0.5778 | 0.0829 | DNA-directed RNA polymerase II subunit RPB1 |
| PF3D7_0402000 | 115195 | 0.2194 | 0.3 | 0.0806 | Plasmodium exported protein (PHISTa), unknown function |
| PF3D7_0404500 | 242143 | 0.3218 | 0.4125 | 0.0907 | 6-cysteine protein P52 |
| PF3D7_0405100 | 276127 | 0.244 | 0.3146 | 0.0706 | protein transport protein Sec24B, putative |
| PF3D7_0405300 | 282524 | 0.3957 | 0.4778 | 0.0821 | liver specific protein 2, putative |
| PF3D7_0405300 | 286547 | 0.3351 | 0.4167 | 0.0815 | liver specific protein 2, putative |
| PF3D7_0405400 | 300851 | 0.2584 | 0.3298 | 0.0714 | pre-mRNA-processing-splicing factor 8, putative |
| PF3D7_0405700 | 306875 | 0.4022 | 0.5 | 0.0978 | lysine decarboxylase, putative |
| PF3D7_0408600 | 415962 | 0.1981 | 0.2842 | 0.0861 | sporozoite invasion-associated protein 1 |
| PF3D7_0409000 | 428626 | 0.502 | 0.6098 | 0.1077 | conserved Plasmodium protein, unknown function |
| PF3D7_0412000 | 538971 | 0.3576 | 0.4659 | 0.1084 | LITAF-like zinc finger protein, putative |
| PF3D7_0412300 | 543724 | 0.372 | 0.4458 | 0.0738 | phosphopantothenoylcysteine synthetase, putative |
| PF3D7_0412300 | 544534 | 0.2642 | 0.3455 | 0.0813 | phosphopantothenoylcysteine synthetase, putative |
| PF3D7_0412300 | 544537 | 0.2512 | 0.3364 | 0.0852 | phosphopantothenoylcysteine synthetase, putative |
| PF3D7_0412300 | 544673 | 0.4915 | 0.5818 | 0.0903 | phosphopantothenoylcysteine synthetase, putative |
| PF3D7_0414100 | 635170 | 0.3909 | 0.481 | 0.0901 | conserved Plasmodium membrane protein, unknown function |
| PF3D7_0418000 | 794319 | 0.4398 | 0.5545 | 0.1147 | conserved Plasmodium protein, unknown function |
| PF3D7_0418000 | 798105 | 0.2562 | 0.3478 | 0.0916 | conserved Plasmodium protein, unknown function |
| PF3D7_0420000 | 899900 | 0.4682 | 0.569 | 0.1008 | zinc finger protein, putative |
| PF3D7_0420000 | 899906 | 0.4682 | 0.569 | 0.1008 | zinc finger protein, putative |
| PF3D7_0420300 | 923964 | 0.1464 | 0.2269 | 0.0805 | AP2 domain transcription factor, putative |
| PF3D7_0420400 | 929521 | 0.463 | 0.534 | 0.071 | ribosome-recycling factor |
| PF3D7_0421700 | 992672 | 0.3363 | 0.4086 | 0.0723 | conserved Plasmodium protein, unknown function |
| PF3D7_0424400 | 1102555 | 0.282 | 0.3544 | 0.0724 | surface-associated interspersed protein 4.2 (SURFIN 4.2) |
| PF3D7_0424400 | 1102558 | 0.2939 | 0.378 | 0.0841 | surface-associated interspersed protein 4.2 (SURFIN 4.2) |
| PF3D7_0424400 | 1102899 | 0.504 | 0.6032 | 0.0991 | surface-associated interspersed protein 4.2 (SURFIN 4.2) |
| PF3D7_0504800 | 194211 | 0.1788 | 0.25 | 0.0712 | conserved Plasmodium protein, unknown function |
| PF3D7_0505000 | 213086 | 0.4872 | 0.6238 | 0.1366 | MMS19-like protein, putative |
| PF3D7_0508000 | 329511 | 0.2792 | 0.3505 | 0.0713 | 6-cysteine protein |
| PF3D7_0508100 | 335910 | 0.3739 | 0.5 | 0.1261 | SET domain protein, putative |
| PF3D7_0509400 | 391061 | 0.3795 | 0.4528 | 0.0733 | RNA polymerase I |
| PF3D7_0511500 | 496535 | 0.0976 | 0.1694 | 0.0717 | RNA pseudouridylate synthase, putative |
| PF3D7_0511500 | 496537 | 0.0976 | 0.1694 | 0.0717 | RNA pseudouridylate synthase, putative |
| PF3D7_0522400 | 929627 | 0.2223 | 0.3214 | 0.0991 | conserved Plasmodium protein, unknown function |
| PF3D7_0526600 | 1113509 | 0.4837 | 0.5556 | 0.0719 | conserved Plasmodium protein, unknown function |
| PF3D7_0528200 | 1164344 | 0.3476 | 0.4286 | 0.081 | eukaryotic translation initiation factor 3 subunit E, putative |
| PF3D7_0532300 | 1308834 | 0.4665 | 0.5882 | 0.1217 | Plasmodium exported protein (PHISTb), unknown function |
| PF3D7_0604400 | 185144 | 0.212 | 0.2826 | 0.0706 | conserved protein, unknown function |
| PF3D7_0605600 | 229484 | 0.4163 | 0.5085 | 0.0922 | nucleoside diphosphate kinase, putative |
| PF3D7_0611800 | 493454 | 0.2926 | 0.3644 | 0.0718 | conserved Plasmodium protein, unknown function |
| PF3D7_0613800 | 577389 | 0.4996 | 0.5882 | 0.0886 | AP2 domain transcription factor, putative |
| PF3D7_0614100 | 592118 | 0.2539 | 0.3333 | 0.0794 | conserved Plasmodium protein, unknown function |
| PF3D7_0619300 | 811025 | 0.2245 | 0.3028 | 0.0782 | conserved Plasmodium protein, unknown function |
| PF3D7_0619600 | 825473 | 0.4223 | 0.5045 | 0.0822 | conserved Plasmodium protein, unknown function |
| PF3D7_0620400 | 852549 | 0.502 | 0.5732 | 0.0712 | merozoite surface protein 10 |
| PF3D7_0621700 | 884526 | 0.3211 | 0.4382 | 0.1171 | conserved Plasmodium protein, unknown function |
| PF3D7_0621700 | 884527 | 0.3211 | 0.4382 | 0.1171 | conserved Plasmodium protein, unknown function |
| PF3D7_0625100 | 1023829 | 0.1454 | 0.2185 | 0.0731 | sphingomyelin synthase 2, putative |
| PF3D7_0627800 | 1117519 | 0.3322 | 0.4268 | 0.0947 | acetyl-CoA synthetase, putative |
| PF3D7_0628100 | 1124425 | 0.4941 | 0.5657 | 0.0716 | HECT-domain (ubiquitin-transferase), putative |
| PF3D7_0628100 | 1128748 | 0.3302 | 0.4327 | 0.1025 | HECT-domain (ubiquitin-transferase), putative |
| PF3D7_0628100 | 1141114 | 0.4548 | 0.5287 | 0.074 | HECT-domain (ubiquitin-transferase), putative |
| PF3D7_0628100 | 1149142 | 0.3424 | 0.425 | 0.0826 | HECT-domain (ubiquitin-transferase), putative |
| PF3D7_0628200 | 1160101 | 0.364 | 0.4444 | 0.0804 | eukaryotic translation initiation factor 2-alpha kinase |
| PF3D7_0629700 | 1224777 | 0.5009 | 0.5851 | 0.0842 | SET domain protein, putative |
| PF3D7_0701900 | 80694 | 0.4904 | 0.5694 | 0.079 | Plasmodium exported protein, unknown function |
| PF3D7_0702000 | 85145 | 0.1654 | 0.2473 | 0.0819 | Plasmodium exported protein (hyp12), unknown function |
| PF3D7_0702000 | 85163 | 0.1669 | 0.25 | 0.0831 | Plasmodium exported protein (hyp12), unknown function |
| PF3D7_0703200 | 126263 | 0.4896 | 0.5747 | 0.0851 | conserved Plasmodium protein, unknown function |
| PF3D7_0703900 | 160439 | 0.4995 | 0.5926 | 0.0931 | conserved Plasmodium membrane protein, unknown function |
| PF3D7_0704600 | 228297 | 0.4975 | 0.6029 | 0.1054 | E3 ubiquitin-protein ligase |
| PF3D7_0707200 | 333500 | 0.4397 | 0.5455 | 0.1058 | conserved Plasmodium protein, unknown function |
| PF3D7_0709300 | 416615 | 0.4765 | 0.5545 | 0.078 | Cg2 protein |
| PF3D7_0709600 | 432784 | 0.4448 | 0.5476 | 0.1028 | ribonucleases P/MRP protein subunit POP1, putative |
| PF3D7_0710000 | 452395 | 0.4047 | 0.48 | 0.0753 | conserved Plasmodium protein, unknown function |
| PF3D7_0710100 | 461399 | 0.2643 | 0.3377 | 0.0734 | conserved protein, unknown function |
| PF3D7_0710200 | 465579 | 0.4514 | 0.5472 | 0.0957 | conserved Plasmodium protein, unknown function |
| PF3D7_0710200 | 469454 | 0.3349 | 0.4384 | 0.1034 | conserved Plasmodium protein, unknown function |
| PF3D7_0711500 | 506750 | 0.5034 | 0.5955 | 0.0921 | regulator of chromosome condensation, putative |
| PF3D7_0713100 | 606298 | 0.1826 | 0.2537 | 0.0711 | Pfmc-2TM Maurer's cleft two transmembrane protein |
| PF3D7_0713500 | 614408 | 0.1929 | 0.2708 | 0.078 | conserved Plasmodium protein, unknown function |
| PF3D7_0713500 | 616290 | 0.1395 | 0.211 | 0.0715 | conserved Plasmodium protein, unknown function |
| PF3D7_0713500 | 616299 | 0.1395 | 0.211 | 0.0715 | conserved Plasmodium protein, unknown function |
| PF3D7_0716300 | 717448 | 0.1513 | 0.2342 | 0.0829 | conserved protein, unknown function |
| PF3D7_0724100 | 1015305 | 0.1001 | 0.1709 | 0.0708 | conserved Plasmodium protein, unknown function |
| PF3D7_0729700 | 1260728 | 0.1739 | 0.2571 | 0.0833 | conserved protein, unknown function |
| PF3D7_0731500 | 1360200 | 0.2673 | 0.3457 | 0.0783 | erythrocyte binding antigen-175 |
| PF3D7_0801700 | 115528 | 0.3323 | 0.4107 | 0.0784 | sentrin-specific protease 2, putative |
| PF3D7_0802000 | 146105 | 0.4718 | 0.5783 | 0.1065 | glutamate dehydrogenase, putative |
| PF3D7_0807700 | 398672 | 0.2866 | 0.3625 | 0.0759 | serine protease DegP |
| PF3D7_0811500 | 582204 | 0.5038 | 0.5972 | 0.0935 | histone-arginine methyltransferase CARM1, putative |
| PF3D7_0811600 | 585841 | 0.4978 | 0.5814 | 0.0836 | conserved protein, unknown function |
| PF3D7_0811600 | 585854 | 0.4711 | 0.5422 | 0.0711 | conserved protein, unknown function |
| PF3D7_0811600 | 586054 | 0.4949 | 0.5909 | 0.096 | conserved protein, unknown function |
| PF3D7_0811800 | 595033 | 0.149 | 0.2203 | 0.0713 | conserved Plasmodium protein, unknown function |
| PF3D7_0818300 | 834628 | 0.4071 | 0.4825 | 0.0754 | dynactin subunit 6, putative |
| PF3D7_0819600 | 885980 | 0.3989 | 0.4775 | 0.0786 | conserved protein, unknown function |
| PF3D7_0826200 | 1143781 | 0.5031 | 0.5978 | 0.0947 | alpha/beta hydrolase, putative |
| PF3D7_0827700 | 1194820 | 0.2808 | 0.3704 | 0.0896 | magnesium transporter, putative |
| PF3D7_0828300 | 1221638 | 0.3899 | 0.4861 | 0.0962 | conserved protein, unknown function |
| PF3D7_0830500 | 1296885 | 0.1095 | 0.1848 | 0.0753 | sporozoite and liver stage tryptophan-rich protein, putative |
| PF3D7_0830800 | 1310862 | 0.3373 | 0.4706 | 0.1333 | surface-associated interspersed protein 8.2 (SURFIN 8.2) |
| PF3D7_0830800 | 1314649 | 0.3639 | 0.4578 | 0.094 | surface-associated interspersed protein 8.2 (SURFIN 8.2) |
| PF3D7_0830900 | 1318079 | 0.2011 | 0.3043 | 0.1032 | Plasmodium exported protein, unknown function |
| PF3D7_0830900 | 1318108 | 0.2091 | 0.3012 | 0.0921 | Plasmodium exported protein, unknown function |
| PF3D7_0831100 | 1325177 | 0.3073 | 0.4028 | 0.0955 | surface-associated interspersed protein 8.1 (SURFIN 8.1) |
| PF3D7_0831100 | 1325187 | 0.4971 | 0.5694 | 0.0724 | surface-associated interspersed protein 8.1 (SURFIN 8.1) |
| PF3D7_0831600 | 1361801 | 0.3235 | 0.4535 | 0.13 | cytoadherence linked asexual protein 8 |
| PF3D7_0831600 | 1362947 | 0.3399 | 0.4125 | 0.0726 | cytoadherence linked asexual protein 9 |
| PF3D7_0831600 | 1362956 | 0.4723 | 0.5455 | 0.0732 | cytoadherence linked asexual protein 10 |
| PF3D7_0902900 | 127688 | 0.2496 | 0.3438 | 0.0942 | conserved Plasmodium protein, unknown function |
| PF3D7_0903600 | 168615 | 0.4 | 0.494 | 0.0939 | conserved protein, unknown function, unspecified product |
| PF3D7_0913900 | 597208 | 0.2302 | 0.3014 | 0.0711 | arginine--tRNA ligase, putative |
| PF3D7_0914300 | 614687 | 0.1221 | 0.1927 | 0.0706 | met-10+ like protein, putative |
| PF3D7_0919900 | 813714 | 0.4919 | 0.5644 | 0.0724 | regulator chromosome condensation-PP1-interacting protein |
| PF3D7_0924600 | 1005351 | 0.43 | 0.5042 | 0.0742 | conserved Plasmodium protein, unknown function |
| PF3D7_0930300 | 1202713 | 0.2881 | 0.3684 | 0.0803 | merozoite surface protein 1 |
| PF3D7_0936300 | 1437244 | 0.3772 | 0.4865 | 0.1093 | ring-exported protein 3 |
| PF3D7_1001300 | 73509 | 0.161 | 0.2376 | 0.0766 | Plasmodium exported protein (PHISTa), unknown function |
| PF3D7_1001700 | 92749 | 0.2898 | 0.3619 | 0.0721 | Plasmodium exported protein (PHISTc), unknown function |
| PF3D7_1004200 | 194771 | 0.1771 | 0.265 | 0.0879 | WD repeat-containing protein, putative |
| PF3D7_1020300 | 821899 | 0.2494 | 0.3248 | 0.0754 | cytoplasmic dynein intermediate chain, putative |
| PF3D7_1022000 | 922261 | 0.3699 | 0.4519 | 0.0821 | RNA-binding protein, putative |
| PF3D7_1029100 | 1191732 | 0.4048 | 0.4752 | 0.0705 | conserved Plasmodium protein, unknown function |
| PF3D7_1030100 | 1228502 | 0.4171 | 0.4894 | 0.0723 | pre-mRNA-splicing factor ATP-dependent RNA helicase |
| PF3D7_1030400 | 1242165 | 0.121 | 0.1927 | 0.0717 | conserved protein, unknown function |
| PF3D7_1031900 | 1285388 | 0.2621 | 0.3478 | 0.0858 | conserved Plasmodium protein, unknown function |
| PF3D7_1033100 | 1327695 | 0.38 | 0.481 | 0.101 | S-adenosylmethionine decarboxylase/ornithine decarboxylase |
| PF3D7_1035100 | 1391800 | 0.2922 | 0.3797 | 0.0876 | probable protein, unknown function |
| PF3D7_1035100 | 1392624 | 0.2322 | 0.3086 | 0.0765 | probable protein, unknown function |
| PF3D7_1038600 | 1551520 | 0.4775 | 0.55 | 0.0725 | Plasmodium exported protein, unknown function |
| PF3D7_1102500 | 120959 | 0.3166 | 0.407 | 0.0903 | Plasmodium exported protein (PHISTb), unknown function |
| PF3D7_1116700 | 632878 | 0.3961 | 0.4677 | 0.0716 | dipeptidyl aminopeptidase 1 |
| PF3D7_1116800 | 637436 | 0.4054 | 0.5 | 0.0946 | heat shock protein 101 |
| PF3D7_1116900 | 644562 | 0.2667 | 0.3425 | 0.0757 | conserved protein, unknown function |
| PF3D7_1120700 | 785839 | 0.0825 | 0.1557 | 0.0732 | conserved Plasmodium protein, unknown function |
| PF3D7_1132500 | 1263156 | 0.377 | 0.4615 | 0.0846 | amino acid transporter, putative |
| PF3D7_1132500 | 1263784 | 0.4696 | 0.5614 | 0.0918 | amino acid transporter, putative |
| PF3D7_1141900 | 1677485 | 0.343 | 0.4167 | 0.0737 | inner membrane complex protein 1b, putative |
| PF3D7_1149600 | 2002782 | 0.1895 | 0.2673 | 0.0778 | DnaJ protein, putative |
| PF3D7_1201400 | 95517 | 0.4569 | 0.5488 | 0.0919 | Plasmodium exported protein, unknown function |
| PF3D7_1201400 | 95535 | 0.1441 | 0.2424 | 0.0983 | Plasmodium exported protein, unknown function |
| PF3D7_1201400 | 95701 | 0.4804 | 0.5875 | 0.1071 | Plasmodium exported protein, unknown function |
| PF3D7_1208100 | 363786 | 0.3659 | 0.4375 | 0.0716 | conserved Plasmodium protein, unknown function |
| PF3D7_1208100 | 372080 | 0.4883 | 0.5631 | 0.0748 | conserved Plasmodium protein, unknown function |
| PF3D7_1208500 | 397531 | 0.1292 | 0.2033 | 0.0741 | conserved Plasmodium protein, unknown function |
| PF3D7_1208500 | 397536 | 0.1292 | 0.2033 | 0.0741 | conserved Plasmodium protein, unknown function |
| PF3D7_1215900 | 643241 | 0.2458 | 0.3162 | 0.0705 | serpentine receptor, putative |
| PF3D7_1219000 | 746752 | 0.3221 | 0.4054 | 0.0833 | formin 2 |
| PF3D7_1219100 | 760531 | 0.5025 | 0.6133 | 0.1108 | clathrin heavy chain, putative |
| PF3D7_1221700 | 866322 | 0.3944 | 0.4655 | 0.0711 | FbpA domain protein, putative |
| PF3D7_1222400 | 892798 | 0.4469 | 0.525 | 0.0781 | AP2 domain transcription factor |
| PF3D7_1239800 | 1660673 | 0.4404 | 0.5122 | 0.0718 | conserved Plasmodium protein, unknown function |
| PF3D7_1239800 | 1670718 | 0.5026 | 0.5882 | 0.0857 | conserved Plasmodium protein, unknown function |
| PF3D7_1248700 | 1998804 | 0.2344 | 0.3103 | 0.0759 | conserved Plasmodium protein, unknown function |
| PF3D7_1250100 | 2054105 | 0.1622 | 0.2609 | 0.0987 | osmiophilic body protein G377 |
| PF3D7_1252400 | 2140526 | 0.501 | 0.5778 | 0.0767 | reticulocyte binding protein homologue 3, pseudogene |
| PF3D7_1301600 | 93019 | 0.2818 | 0.36 | 0.0782 | erythrocyte binding antigen-140 |
| PF3D7_1303800 | 211721 | 0.4736 | 0.5625 | 0.0889 | conserved Plasmodium protein, unknown function |
| PF3D7_1306500 | 305931 | 0.4842 | 0.578 | 0.0938 | MORN repeat protein, putative |
| PF3D7_1308400 | 388365 | 0.3324 | 0.4074 | 0.075 | conserved Plasmodium protein, unknown function |
| PF3D7_1312200 | 518278 | 0.1612 | 0.2456 | 0.0844 | conserved protein, unknown function |
| PF3D7_1312800 | 542106 | 0.2492 | 0.3363 | 0.087 | conserved Plasmodium protein, unknown function |
| PF3D7_1314800 | 629898 | 0.4771 | 0.5584 | 0.0813 | ubiquitin-like protein, putative |
| PF3D7_1315100 | 639213 | 0.5014 | 0.5943 | 0.0929 | serine/threonine protein kinase PK9 |
| PF3D7_1315700 | 659516 | 0.3464 | 0.419 | 0.0726 | tRNA (adenine(58)-N(1))-methyltransferase catalytic subunit |
| PF3D7_1320700 | 853970 | 0.4667 | 0.5789 | 0.1122 | conserved Plasmodium protein, unknown function |
| PF3D7_1321900 | 911843 | 0.3266 | 0.4286 | 0.102 | conserved protein, unknown function |
| PF3D7_1335100 | 1419415 | 0.3722 | 0.4494 | 0.0772 | merozoite surface protein 7 |
| PF3D7_1335100 | 1419420 | 0.4226 | 0.5111 | 0.0885 | merozoite surface protein 8 |
| PF3D7_1335100 | 1419427 | 0.3241 | 0.3958 | 0.0717 | merozoite surface protein 9 |
| PF3D7_1335900 | 1466264 | 0.1544 | 0.2258 | 0.0714 | thrombospondin-related anonymous protein |
| PF3D7_1335900 | 1466371 | 0.4337 | 0.5694 | 0.1358 | thrombospondin-related anonymous protein |
| PF3D7_1342200 | 1661966 | 0.4474 | 0.5258 | 0.0784 | conserved Plasmodium membrane protein, unknown function |
| PF3D7_1347200 | 1890723 | 0.4403 | 0.5128 | 0.0725 | nucleoside transporter 1 |
| PF3D7_1347200 | 1891015 | 0.4325 | 0.5062 | 0.0737 | conserved Plasmodium membrane protein, unknown function |
| PF3D7_1349400 | 1984018 | 0.4658 | 0.5362 | 0.0704 | cytidine and deoxycytidylate deaminase, putative |
| PF3D7_1351200 | 2043725 | 0.3745 | 0.4694 | 0.0949 | conserved Plasmodium protein, unknown function |
| PF3D7_1352700 | 2102174 | 0.464 | 0.5577 | 0.0936 | intron-binding protein aquarius, putative |
| PF3D7_1354600 | 2176530 | 0.1636 | 0.2632 | 0.0996 | 60S ribosomal protein L7-2, putative |
| PF3D7_1360500 | 2417849 | 0.2305 | 0.3298 | 0.0993 | guanylyl cyclase beta |
| PF3D7_1360800 | 2435370 | 0.2647 | 0.3563 | 0.0916 | falcilysin |
| PF3D7_1361800 | 2481275 | 0.2072 | 0.2889 | 0.0817 | glideosome-associated connector |
| PF3D7_1365300 | 2614473 | 0.5029 | 0.5922 | 0.0893 | conserved Plasmodium protein, unknown function |
| PF3D7_1366700 | 2666855 | 0.4766 | 0.549 | 0.0724 | conserved Plasmodium protein, unknown function |
| PF3D7_1367900 | 2708270 | 0.244 | 0.3182 | 0.0742 | conserved Plasmodium protein, unknown function |
| PF3D7_1409500 | 370534 | 0.2353 | 0.3301 | 0.0948 | conserved Plasmodium protein, unknown function |
| PF3D7_1409600 | 375126 | 0.2207 | 0.3153 | 0.0946 | conserved Plasmodium protein, unknown function |
| PF3D7_1410000 | 393502 | 0.481 | 0.5532 | 0.0722 | ER membrane protein complex subunit 2, putative |
| PF3D7_1430400 | 1202249 | 0.2769 | 0.3516 | 0.0748 | autophagy protein 5, putative |
| PF3D7_1433500 | 1334365 | 0.4625 | 0.5385 | 0.076 | DNA topoisomerase 2 |
| PF3D7_1434500 | 1386651 | 0.1454 | 0.2167 | 0.0713 | dynein-related AAA-type ATPase, putative |
| PF3D7_1434500 | 1395386 | 0.3194 | 0.3913 | 0.0719 | dynein-related AAA-type ATPase, putative |
| PF3D7_1436300 | 1480704 | 0.2487 | 0.3261 | 0.0774 | translocon component PTEX150 |
| PF3D7_1436300 | 1480705 | 0.2396 | 0.3111 | 0.0715 | translocon component PTEX151 |
| PF3D7_1440900 | 1669663 | 0.309 | 0.382 | 0.0731 | conserved Plasmodium protein, unknown function |
| PF3D7_1451900 | 2128849 | 0.1424 | 0.2547 | 0.1123 | ribosome biogenesis protein TSR1, putative |
| PF3D7_1452700 | 2169240 | 0.3148 | 0.4035 | 0.0887 | U1 snRNP-associated protein, putative |
| PF3D7_1454900 | 2245138 | 0.6545 | 0.7353 | 0.0808 | conserved protein, unknown function |
| PF3D7_1456000 | 2296880 | 0.2527 | 0.3306 | 0.0779 | AP2 domain transcription factor, putative |
| PF3D7_1457400 | 2355803 | 0.2563 | 0.3448 | 0.0885 | conserved Plasmodium protein, unknown function |
| PF3D7_1464600 | 2621742 | 0.3613 | 0.4457 | 0.0844 | serine/threonine protein phosphatase UIS2, putative |
| PF3D7_1465800 | 2672397 | 0.0712 | 0.1455 | 0.0742 | dynein beta chain, putative |
| PF3D7_1471600 | 2929017 | 0.3061 | 0.3772 | 0.0711 | conserved Plasmodium protein, unknown function |
| PF3D7_1473700 | 2999459 | 0.3816 | 0.4712 | 0.0895 | nucleoporin NUP116/NSP116, putative |
| PF3D7_1475900 | 3124462 | 0.2396 | 0.3125 | 0.0729 | KELT protein |
| PF3D7_1475900 | 3124586 | 0.4121 | 0.4828 | 0.0706 | KELT protein |
| PF3D7_1475900 | 3125454 | 0.5042 | 0.6182 | 0.114 | KELT protein |
| PF3D7_1475900 | 3125463 | 0.1321 | 0.2292 | 0.0971 | KELT protein |
| PF3D7_1475900 | 3126075 | 0.4804 | 0.5972 | 0.1168 | KELT protein |

*****Proportion of mismatches within individuals minus the proportion of mismatches between individuals.
