# Supplementary material for: Whole-genome analysis of Malawian Plasmodium falciparum isolates identifies possible targets of allele-specific immunity to clinical malaria
Source: PLoS Genet. 2021 May 25;17(5):e1009576. doi: 10.1371/journal.pgen.1009576 (PMC8184011; doi:10.1371/journal.pgen.1009576)
Supplement: S3 Table — (DOCX) [file pgen.1009576.s008.docx]

| **S3 Table.** **Heatmap of Fragments Per Kilobase of transcript per Million mapped reads (FPKM) values to show expression of 25 identified genes during the intraerythrocytic stages of the parasite life cycle*** [46]. Warmer colors indicate lower expression. | | | | | | | | | |
| --- | --- | --- | --- | --- | --- | --- | --- | --- | --- |
|  | **Hours Post Invasion** | | | | | | | | |
| **Gene** | **T05** | **T10** | **T15** | **T20** | **T25** | **T30** | **T35** | **T40** | **T45** |
| PF3D7_0311900 |  |  |  |  |  |  |  |  |  |
| PF3D7_0312500 |  |  |  |  |  |  |  |  |  |
| PF3D7_0318200 |  |  |  |  |  |  |  |  |  |
| PF3D7_0412300 |  |  |  |  |  |  |  |  |  |
| PF3D7_0421700 |  |  |  |  |  |  |  |  |  |
| PF3D7_0424400 |  |  |  |  |  |  |  |  |  |
| PF3D7_0511500 |  |  |  |  |  |  |  |  |  |
| PF3D7_0522400 |  |  |  |  |  |  |  |  |  |
| PF3D7_0526600 |  |  |  |  |  |  |  |  |  |
| PF3D7_0605600 |  |  |  |  |  |  |  |  |  |
| PF3D7_0619600 |  |  |  |  |  |  |  |  |  |
| PF3D7_0704600 |  |  |  |  |  |  |  |  |  |
| PF3D7_0710200 |  |  |  |  |  |  |  |  |  |
| PF3D7_0807700 |  |  |  |  |  |  |  |  |  |
| PF3D7_0831600 |  |  |  |  |  |  |  |  |  |
| PF3D7_0914300 |  |  |  |  |  |  |  |  |  |
| PF3D7_1004200 |  |  |  |  |  |  |  |  |  |
| PF3D7_1030400 |  |  |  |  |  |  |  |  |  |
| PF3D7_1033100 |  |  |  |  |  |  |  |  |  |
| PF3D7_1035100 |  |  |  |  |  |  |  |  |  |
| PF3D7_1102500 |  |  |  |  |  |  |  |  |  |
| PF3D7_1149600 |  |  |  |  |  |  |  |  |  |
| PF3D7_1219100 |  |  |  |  |  |  |  |  |  |
| PF3D7_1465800 |  |  |  |  |  |  |  |  |  |
| PF3D7_1475900 |  |  |  |  |  |  |  |  |  |
| **FPKM Values Scale**: | 0 | 250 | 500 | 750 | 1000 | 1250 | 1500 | 1750 | 2556 |
| *T05 indicates the early ring stage of the parasite life-cycle, immediately after the invasion of the red blood cell by merozoites. The trophozoite stage of the intraerythrocyte cycle begins around approximately 24 hours post invasion (T25) and the schizong stage begins around 40 hours post invasion (T40). | | | | | | | | | |
