# Supplementary material for: Whole-genome analysis of Malawian Plasmodium falciparum isolates identifies possible targets of allele-specific immunity to clinical malaria
Source: PLoS Genet. 2021 May 25;17(5):e1009576. doi: 10.1371/journal.pgen.1009576 (PMC8184011; doi:10.1371/journal.pgen.1009576)
Supplement: S4 Table — (DOCX) [file pgen.1009576.s009.docx]

| **S4 Table. Protein/Gene features for genes identified with both analytical approaches** [47]**.** | | | | | | | | |
| --- | --- | --- | --- | --- | --- | --- | --- | --- |
|  | **Polymorphism** | **Essentiality** | | **Transmembrane (TM)/ Signal Peptides** | | **Protein Expression**  **(Global proteome and phosphoproteome)** | | |
| **Gene ID** | **NonSyn/Syn SNP Ratio** | **Mutagenesis Index Score** | **Mutant fitness score** | **# TM Domains** | **Signal Peptide** | **Ring** | **Troph** | **Schizont** |
| PF3D7_0311900 | 6.2 | 0.12 | -3.078 | 1 | null | null | null | null |
| PF3D7_0312500 | 2.11 | 1 | -1.543 | 12 | null | 3.91 | 4.39 | 3.81 |
| PF3D7_0318200 | 2.17 | 0.144 | -2.838 | 0 | null | 4.27 | 3.66 | 4.13 |
| PF3D7_0412300 | 3.22 | 0.98 | -2.835 | 0 | null | 4.25 | 4.09 | 3.81 |
| PF3D7_0421700 | 2.75 | 1 | -0.866 | 0 | null | null | null | null |
| PF3D7_0424400 | 9.2 | 1 | -1.171 | 0 | null | null | null | null |
| PF3D7_0511500 | 2.97 | 1 | -1.927 | 0 | null | 4.25 | 4.09 | 3.81 |
| PF3D7_0522400 | 3.36 | 1 | -2.985 | 2 | null | 4.46 | 3.81 | 3.81 |
| PF3D7_0526600 | 3.5 | 1 | -1.03 | 0 | null | null | null | null |
| PF3D7_0605600 | 3.65 | 1 | -1.684 | 0 | null | null | null | null |
| PF3D7_0619600 | 3.17 | 0.148 | -2.836 | 2 | null | null | null | null |
| PF3D7_0704600 | 4.32 | 1 | -2.305 | 4 | null | 4.25 | 3.91 | 4 |
| PF3D7_0710200 | 4.13 | 0.153 | -2.841 | 0 | null | 4.52 | 4.17 | 3.17 |
| PF3D7_0807700 | 6.06 | 1 | 0.991 | 0 | Yes | null | null | null |
| PF3D7_0831600 | 3.31 | 1 | -0.705 | 0 | Yes | 4.17 | 3.81 | 4.17 |
| PF3D7_0914300 | 6 | 1 | -0.616 | 1 | Yes | 4.25 | 4.09 | 3.81 |
| PF3D7_1004200 | 2.98 | 0.193 | -2.75 | 4 | null | 4.46 | 3.91 | 3.7 |
| PF3D7_1030400 | 8 | 1 | -0.726 | 0 | null | null | null | null |
| PF3D7_1033100 | 4.4 | 0.18 | -2.799 | 0 | null | 4.46 | 4.09 | 3.46 |
| PF3D7_1035100 | 3.69 | 0.925 | -2.293 | 0 | Yes | null | null | null |
| PF3D7_1102500 | 12.33 | 0.234 | -2.787 | 1 | null | 3.7 | 4 | 4.39 |
| PF3D7_1149600 | 3.33 | 1 | -0.493 | 1 | null | 3.7 | 4.25 | 4.09 |
| PF3D7_1219100 | 1.32 | 0.121 | -3.091 | 0 | null | 4.39 | 4.09 | 3.58 |
| PF3D7_1465800 | 2.78 | 1 | -1.429 | 0 | null | null | null | null |
| PF3D7_1475900 | 15.86 | 1 | -0.703 | 0 | null | null | null | null |
