# Supplementary material for: Whole-genome analysis of Malawian Plasmodium falciparum isolates identifies possible targets of allele-specific immunity to clinical malaria
Source: PLoS Genet. 2021 May 25;17(5):e1009576. doi: 10.1371/journal.pgen.1009576 (PMC8184011; doi:10.1371/journal.pgen.1009576)
Supplement: S5 Table — (DOCX) [file pgen.1009576.s010.docx]

| **S5 Table. Pairwise genetic differentiation (F_ST_) between clag8 sequences from Africa, Asia and Papua New Guinea (PNG).** | | | |
| --- | --- | --- | --- |
|  | **Africa** | **Asia** | **PNG** |
| **Africa** | - | 0.002 | 0.003 |
| **Asia** |  | - | 0.003 |
| **PNG** |  |  | - |
