# Supplementary material for: Whole-genome analysis of Malawian Plasmodium falciparum isolates identifies possible targets of allele-specific immunity to clinical malaria
Source: PLoS Genet. 2021 May 25;17(5):e1009576. doi: 10.1371/journal.pgen.1009576 (PMC8184011; doi:10.1371/journal.pgen.1009576)
Supplement: S6 Table — (DOCX) [file pgen.1009576.s011.docx]

**S6 Table. NCBI accession numbers.**

| **NCBI ID** | **BioProject** | **BioSample** |
| --- | --- | --- |
| IGS-MWI-006sA | PRJNA312679 | SAMN08815771 |
| IGS-MWI-113sA | PRJNA447987 | SAMN08815631 |
| IGS-MWI-008sA | PRJNA312679 | SAMN08815773 |
| IGS-MWI-116sA | PRJNA447987 | SAMN08815634 |
| IGS-MWI-117sA | PRJNA447987 | SAMN08815635 |
| IGS-MWI-118sA | PRJNA447987 | SAMN08815636 |
| IGS-MWI-010sA | PRJNA312679 | SAMN08815775 |
| IGS-MWI-011sA | PRJNA312679 | SAMN08815776 |
| IGS-MWI-012sA | PRJNA312679 | SAMN08815777 |
| IGS-MWI-013sA | PRJNA312679 | SAMN08815778 |
| IGS-MWI-119sA | PRJNA447987 | SAMN08815637 |
| IGS-MWI-120sA | PRJNA447987 | SAMN08815638 |
| IGS-MWI-015sA | PRJNA312679 | SAMN08815780 |
| IGS-MWI-122sA | PRJNA447987 | SAMN08815640 |
| IGS-MWI-124sA | PRJNA447987 | SAMN08815642 |
| IGS-MWI-017sA | PRJNA312679 | SAMN08815782 |
| IGS-MWI-018sA | PRJNA312679 | SAMN08815783 |
| IGS-MWI-019sA | PRJNA312679 | SAMN08815784 |
| IGS-MWI-020sA | PRJNA312679 | SAMN08815785 |
| IGS-MWI-023sA | PRJNA312679 | SAMN08815788 |
| IGS-MWI-125sA | PRJNA447987 | SAMN08815643 |
| IGS-MWI-126sA | PRJNA447987 | SAMN08815644 |
| IGS-MWI-127sA | PRJNA447987 | SAMN08815645 |
| IGS-MWI-025sA | PRJNA312679 | SAMN08815790 |
| IGS-MWI-129sA | PRJNA447987 | SAMN08815647 |
| IGS-MWI-130sA | PRJNA447987 | SAMN08815648 |
| IGS-MWI-026sA | PRJNA312679 | SAMN08815791 |
| IGS-MWI-027sA | PRJNA312679 | SAMN08815792 |
| IGS-MWI-028sA | PRJNA312679 | SAMN08815793 |
| IGS-MWI-131sA | PRJNA447987 | SAMN08815649 |
| IGS-MWI-132sA | PRJNA447987 | SAMN08815650 |
| IGS-MWI-133sA | PRJNA447987 | SAMN08815651 |
| IGS-MWI-029sA | PRJNA312679 | SAMN08815794 |
| IGS-MWI-134sA | PRJNA447987 | SAMN08815652 |
| IGS-MWI-031sA | PRJNA312679 | SAMN08815796 |
| IGS-MWI-032sA | PRJNA312679 | SAMN08815797 |
| IGS-MWI-136sA | PRJNA447987 | SAMN08815654 |
| IGS-MWI-137sA | PRJNA447987 | SAMN08815655 |
| IGS-MWI-138sA | PRJNA447987 | SAMN08815656 |
| IGS-MWI-034sA | PRJNA312679 | SAMN08815799 |
| IGS-MWI-139sA | PRJNA447987 | SAMN08815657 |
| IGS-MWI-140sA | PRJNA447987 | SAMN08815658 |
| IGS-MWI-142sA | PRJNA447987 | SAMN08815660 |
| IGS-MWI-143sA | PRJNA447987 | SAMN08815661 |
| IGS-MWI-144sA | PRJNA447987 | SAMN08815662 |
| IGS-MWI-036sA | PRJNA312679 | SAMN08815801 |
| IGS-MWI-145sA | PRJNA447987 | SAMN08815663 |
| IGS-MWI-146sA | PRJNA447987 | SAMN08815664 |
| IGS-MWI-038sA | PRJNA312679 | SAMN08815803 |
| IGS-MWI-147sA | PRJNA447987 | SAMN08815665 |
| IGS-MWI-040sA | PRJNA312679 | SAMN08815805 |
| IGS-MWI-041sA | PRJNA312679 | SAMN08815806 |
| IGS-MWI-042sA | PRJNA312679 | SAMN08815807 |
| IGS-MWI-043sA | PRJNA312679 | SAMN08815808 |
| IGS-MWI-148sA | PRJNA447987 | SAMN08815666 |
| IGS-MWI-149sA | PRJNA447987 | SAMN08815667 |
| IGS-MWI-045sA | PRJNA312679 | SAMN08815810 |
| IGS-MWI-150sA | PRJNA447987 | SAMN08815668 |
| IGS-MWI-151sA | PRJNA447987 | SAMN08815669 |
| IGS-MWI-046sA | PRJNA312679 | SAMN08815811 |
| IGS-MWI-047sA | PRJNA312679 | SAMN08815812 |
| IGS-MWI-152sA | PRJNA447987 | SAMN08815670 |
| IGS-MWI-049sA | PRJNA312679 | SAMN08815814 |
| IGS-MWI-155sA | PRJNA447987 | SAMN08815673 |
| IGS-MWI-156sA | PRJNA447987 | SAMN08815674 |
| IGS-MWI-157sA | PRJNA447987 | SAMN08815675 |
| IGS-MWI-158sA | PRJNA447987 | SAMN08815676 |
| IGS-MWI-053sA | PRJNA312679 | SAMN08815818 |
| IGS-MWI-055sA | PRJNA312679 | SAMN08815820 |
| IGS-MWI-160sA | PRJNA447987 | SAMN08815678 |
| IGS-MWI-161sA | PRJNA447987 | SAMN08815679 |
| IGS-MWI-163sA | PRJNA447987 | SAMN08815681 |
| IGS-MWI-056sA | PRJNA312679 | SAMN08815821 |
| IGS-MWI-164sA | PRJNA447987 | SAMN08815682 |
| IGS-MWI-057sA | PRJNA312679 | SAMN08815822 |
| IGS-MWI-058sA | PRJNA312679 | SAMN08815823 |
| IGS-MWI-059sA | PRJNA312679 | SAMN08815824 |
| IGS-MWI-167sA | PRJNA447987 | SAMN08815685 |
| IGS-MWI-061sA | PRJNA312679 | SAMN08815826 |
| IGS-MWI-168sA | PRJNA447987 | SAMN08815686 |
| IGS-MWI-169sA | PRJNA447987 | SAMN08815687 |
| IGS-MWI-064sA | PRJNA312679 | SAMN08815829 |
| IGS-MWI-172sA | PRJNA447987 | SAMN08815690 |
| IGS-MWI-068sA | PRJNA312679 | SAMN08815833 |
| IGS-MWI-069sA | PRJNA312679 | SAMN08815834 |
| IGS-MWI-070sA | PRJNA312679 | SAMN08815835 |
| IGS-MWI-173sA | PRJNA447987 | SAMN08815691 |
| IGS-MWI-174sA | PRJNA447987 | SAMN08815692 |
| IGS-MWI-071sA | PRJNA312679 | SAMN08815836 |
| IGS-MWI-072sA | PRJNA312679 | SAMN08815837 |
| IGS-MWI-175sA | PRJNA447987 | SAMN08815693 |
| IGS-MWI-176sA | PRJNA447987 | SAMN08815694 |
| IGS-MWI-177sA | PRJNA447987 | SAMN08815695 |
| IGS-MWI-075sA | PRJNA312679 | SAMN08815840 |
| IGS-MWI-076sA | PRJNA312679 | SAMN08815841 |
| IGS-MWI-178sA | PRJNA447987 | SAMN08815696 |
| IGS-MWI-078sA | PRJNA312679 | SAMN08815843 |
| IGS-MWI-179sA | PRJNA447987 | SAMN08815697 |
| IGS-MWI-180sA | PRJNA447987 | SAMN08815698 |
| IGS-MWI-182sA | PRJNA447987 | SAMN08815700 |
| IGS-MWI-183sA | PRJNA447987 | SAMN08815701 |
| IGS-MWI-080sA | PRJNA312679 | SAMN08815845 |
| IGS-MWI-081sA | PRJNA312679 | SAMN08815846 |
| IGS-MWI-082sA | PRJNA312679 | SAMN08815847 |
| IGS-MWI-083sA | PRJNA312679 | SAMN08815848 |
| IGS-MWI-189sA | PRJNA447987 | SAMN08815707 |
| IGS-MWI-085sA | PRJNA312679 | SAMN08815850 |
| IGS-MWI-191sA | PRJNA447987 | SAMN08815709 |
| IGS-MWI-086sA | PRJNA312679 | SAMN08815851 |
| IGS-MWI-195sA | PRJNA447987 | SAMN08815713 |
| IGS-MWI-090sA | PRJNA312679 | SAMN08815855 |
| IGS-MWI-091sA | PRJNA312679 | SAMN08815856 |
| IGS-MWI-092sA | PRJNA312679 | SAMN08815857 |
| IGS-MWI-196sA | PRJNA447987 | SAMN08815714 |
| IGS-MWI-197sA | PRJNA447987 | SAMN08815715 |
| IGS-MWI-094sA | PRJNA312679 | SAMN08815859 |
| IGS-MWI-198sA | PRJNA447987 | SAMN08815716 |
| IGS-MWI-095sA | PRJNA312679 | SAMN08815860 |
| IGS-MWI-096sA | PRJNA312679 | SAMN08815861 |
| IGS-MWI-199sA | PRJNA447987 | SAMN08815717 |
| IGS-MWI-200sA | PRJNA447987 | SAMN08815718 |
| IGS-MWI-202sA | PRJNA447987 | SAMN08815720 |
| IGS-MWI-203sA | PRJNA447987 | SAMN08815721 |
| IGS-MWI-099sA | PRJNA312679 | SAMN08815864 |
| IGS-MWI-100sA | PRJNA312679 | SAMN08815865 |
| IGS-MWI-101sA | PRJNA312679 | SAMN08815866 |
| IGS-MWI-102sA | PRJNA312679 | SAMN08815867 |
| IGS-MWI-103sA | PRJNA312679 | SAMN08815868 |
| IGS-MWI-204sA | PRJNA447987 | SAMN08815722 |
| IGS-MWI-205sA | PRJNA447987 | SAMN08815723 |
| IGS-MWI-206sA | PRJNA447987 | SAMN08815724 |
| IGS-MWI-207sA | PRJNA447987 | SAMN08815725 |
| IGS-MWI-208sA | PRJNA447987 | SAMN08815726 |
| IGS-MWI-209sA | PRJNA447987 | SAMN08815727 |
| IGS-MWI-213sA | PRJNA447987 | SAMN08815731 |
| IGS-MWI-216sA | PRJNA447987 | SAMN08815734 |
| IGS-MWI-217sA | PRJNA447987 | SAMN08815735 |
| IGS-MWI-218sA | PRJNA447987 | SAMN08815736 |
| IGS-MWI-219sA | PRJNA447987 | SAMN08815737 |
| IGS-MWI-222sA | PRJNA447987 | SAMN08815740 |
